# High-Density Transcriptional Initiation Signals Underline Genomic Islands in Bacteria

**DOI:** 10.1371/journal.pone.0033759

**Published:** 2012-03-20

**Authors:** Qianli Huang, Xuanjin Cheng, Man Kit Cheung, Sergey S. Kiselev, Olga N. Ozoline, Hoi Shan Kwan

**Affiliations:** 1 School of Life Sciences, The Chinese University of Hong Kong, Hong Kong SAR, China; 2 Institute of Cell Biophysics, Russian Academy of Sciences, Moscow, Russia; Niels Bohr Institute, Denmark

## Abstract

Genomic islands (GIs), frequently associated with the pathogenicity of bacteria and having a substantial influence on bacterial evolution, are groups of “alien” elements which probably undergo special temporal–spatial regulation in the host genome. Are there particular hallmark transcriptional signals for these “exotic” regions? We here explore the potential transcriptional signals that underline the GIs beyond the conventional views on basic sequence composition, such as codon usage and GC property bias. It showed that there is a significant enrichment of the transcription start positions (TSPs) in the GI regions compared to the whole genome of *Salmonella enterica* and *Escherichia coli*. There was up to a four-fold increase for the 70% GIs, implying high-density TSPs profile can potentially differentiate the GI regions. Based on this feature, we developed a new sliding window method GIST, **G**enomic-island **I**dentification by **S**ignals of **T**ranscription, to identify these regions. Subsequently, we compared the known GI-associated features of the GIs detected by GIST and by the existing method Islandviewer to those of the whole genome. Our method demonstrates high sensitivity in detecting GIs harboring genes with biased GI-like function, preferred subcellular localization, skewed GC property, shorter gene length and biased “non-optimal” codon usage. The special transcriptional signals discovered here may contribute to the coordinate expression regulation of foreign genes. Finally, by using GIST, we detected many interesting GIs in the 2011 German *E. coli* O104:H4 outbreak strain TY-2482, including the microcin H47 system and gene cluster *ycgXEFZ-ymgABC* that activates the production of biofilm matrix. The aforesaid findings highlight the power of GIST to predict GIs with distinct intrinsic features to the genome. The heterogeneity of cumulative TSPs profiles may not only be a better identity for “alien” regions, but also provide hints to the special evolutionary course and transcriptional regulation of GI regions.

## Introduction

Since the publication of the first pathogenicity island (PAI) [1], originally described as clusters of virulence genes that were identified in uropathogenic *E. coli* but absent in closely related strains, various types of other islands, such as secretion islands and resistance islands, have been detected. The more general term “genomic island” (GI) was then defined as horizontally acquired genomic regions that undergo broad modes of transmission and integration [2]–[6]. Through many studies, GIs have been noted for their important roles in conferring multidrug resistance and pathogenesis [7]–[13]. Recently, more and more important adaptive functions derived from different GIs have been discovered. For instance, it was reported that the emergence of symbiosis is driven by GI [14]. For metabolism, the formation of a new pathway for pollutant degradation is mediated by GI in *Ralstonia* [15], and the GIs are related not only to secondary metabolism but also to primary metabolism in many pathogens [16], [17]. Moreover, the intracellular synthesis of magnetosome, which accumulates ions from aquatic environments, is also conveyed by the GI [18]. Probably, because GIs have multiple important contributions to adaptability, metabolic versatility, fitness and so on [19], a pending problem which attracts continual interests is whether there are any signals which best identify these genomic regions.

So far, many GI-associated sequence features have been utilized to identify GIs *in silico*. According to the theory that different genomes have different preferences in codon usage, codon usage bias was used as a measurement to detect GI by the software SIGI-HMM [20]. Another method, IslandPath-DIMoB, was initially developed based on dinucleotide bias and the association of novel genes with GI [21], and it was lately combined with the SIGI-HMM on the platform Islandviewer [22]. Whereas these methods detect composition heterogeneity within the unit of a single gene or a group of genes, several other methods, such as Alien_Hunter, Centroid and the “top-down” approach reported by Arvey *et al*. [23]–[25], split genomes into progressively smaller regions. Apart from the composition features, including GC content, codon usage and k-mer size bias (which can be of 2–8 nucleotides), many other programs, based on comparative genomics or phylogenic relationship, were also designed to predict mobile alien regions. For instance, MobilomeFINDER identifies GI through searching flanking regions of orthologous tRNA genes, and Yoon *et al*. searched for the presence of homolog(s) of virulence genes to detect PAI regions, and, recently, genomic barcode has also been applied to identify PAIs [26]–[28].

Unfortunately, the performance of the existing methods, irrespective of the feature or phylogenic relationship on which they are based, falls far short of the level which is demanded. For example, although SIGI-HMM shows the highest overall accuracy (about 86%), the recall rate is only 33%. Conversely, Alien_Hunter has a comparatively higher recall rate (about 77%), but this is greatly at the expense of accuracy (only 38%) [29]. Divergent outputs may be mainly caused by the fact that these prediction methods are based on dissimilar sequence composition features or different genome collections for building phylogenic relationships. These imply that there is a serious need for new signatures or signals to serve as benchmarks for the identification of GIs.

Here, we attempt to characterize GIs at a novel dimension – the potential signature of transcription signals. In prokaryotes, gene expression is considered to be controlled mainly at the level of transcription initiation by the repressor or activator proteins. To maximize bacterial fitness and prevent possible detrimental effects resulting from the expression of exotic genes, it is reasonable that GI genes tend to experience more complex regulation than the ancestral genes [30]. So, it is possible that GI regions can be distinguished by the heterogeneity of certain transcription initiation signals. Although many experiments have been designed to disclose the particular transcriptional regulation mechanisms of individual GIs or of single secretion systems [31]–[33], there is no report to detect the special transcriptional regulation signals which can delineate the GIs on genome-wide scale.

In this study, we systematically identified the main promoter attribute – transcription start points (TSPs) across nine *Salmonella enterica* genomes. Strikingly, for all the genomes examined, there is a significant enrichment of potential TSPs in the GI regions. We also found significantly more TSPs in the GI regions of *E. coli* K-12 MG1655, and this demonstrates that the phenomenon is not confined to *Salmonella enterica*. Further, we used a set of objective criteria based on this feature to predict putative GIs on a genome-wide scale. Through the comparative analysis of known features associated with GIs and the application in analyzing the GIs of the German *E. coli* O104:H4 outbreak strain TY-2482, we found that our method is powerful in detecting GI-like regions. From these analyses, we propose that the particular characteristic, significantly more prevalent TSPs in GIs, can manifest the GI, and this signal may sustain the appropriate temporal–spatial transcription of GIs as alien elements in the host cells.

## Results and Discussion

### High-density transcriptional initiation signals associated with GIs

**Figure 1:**
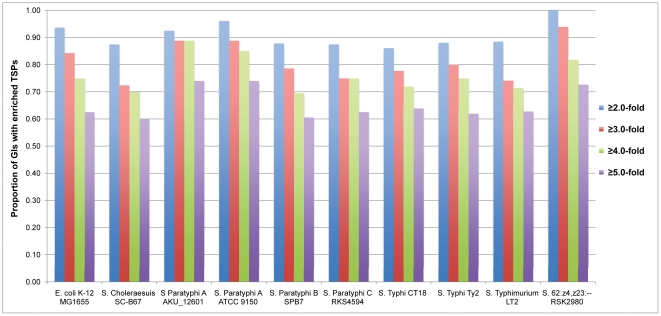
Proportion of GIs with enriched TSPs among ten bacterial genomes. The y-axis represents the proportion of GI regions, and names of bacterial genomes are shown along the x-axis.

To explore potential transcriptional signals related to GIs, we first located promoter sites at the genome-wide level by using the software PlatProm, which has high precision and a low rate of false positives because it exactly inspects the transcription start points (TSPs), rather than defines the extensive promoter regions, through scanning the promoter-specific elements in proper positions on both strands [34] (see Materials and Methods). Thus, relatively weak transcription initiation signals, possibly required for provisional transcripts such as short RNAs or antisense RNAs, will also be recognized [see Datasets S1, 2, 3, 4, 5]. Then, we created a dataset including the GIs detected by various methods in all examined genomes (see Materials and Methods, Figure S1). The length of GI ranges from 4 kb to more than 100 kb and the number of genes in each detected island ranges from 4 to more than 30, reflecting a high diversity in ordinary GI features. To facilitate comparative analysis, the TSPs were matched to the GI regions. Subsequently, we compared the corresponding transcription initiation signals, here denoted by the TSPs, between the GI regions and genomes of the nine *Salmonella* strains. As depicted in Figure 1, more than 88% of the GI regions have a TSP density higher than two-fold of the genome average and the percentage is even higher than 70% when the fold difference is up to four in all examined strains, and even up to five in strains such as *S.* Paratyphi A ATCC 9150, *S.* 62:z4,z23: – RSK2980 and *S.* Paratyphi A AKU_12601. To further check whether this phenomenon is specific to *Salmonella*, the same analysis was performed in *E. coli* K-12 MG1655. Interestingly, similar results were observed (see Figure 1). These suggest that the TSPs are significantly enriched in the GI regions, potentially hinting at a more complex regulation of the GI's transcription initiation.

### Predict the potential novel GIs through GIST: Genomic-island Identification by Signal of Transcription

**Figure 2:**
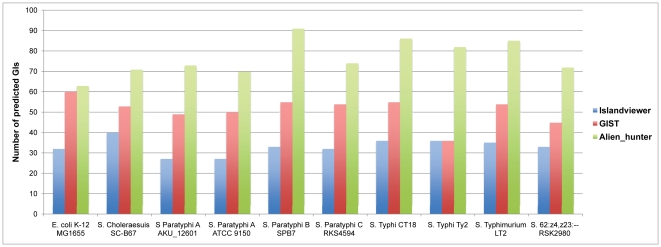
Number of GIs predicted by Islandviewer, GIST and Alien_hunter among ten bacterial genomes. The y-axis represents the number of GIs detected by the three methods, and names of bacterial genomes are shown along the x-axis.

**Figure 3:**
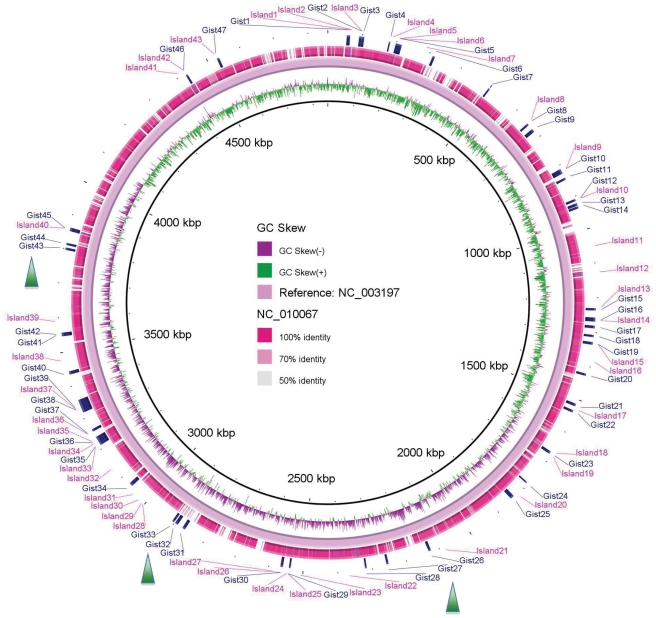
BRIG diagram showing results of GIST and Islandviewer on *Salmonella enterica* 62:z4,z23:– RSK2980 (NC_010067). GIs predicted with Islandviewer are marked as Island1, Island2, etc.; and those predicted by GIST are denoted with Gist1, Gist2, etc. The three main divergent regions detected by GIST but missed by Islandviewer are labeled with green triangles.

To detect the regions with heterogeneous transcriptional initiation signals against the genomes, we have developed a method named “GIST” based on successive windows (see Materials and Methods). Regions with a TSP density 1.5 times higher than a whole genome are selected, and there are 186 regions detected in each genome on average. The detected regions almost include all the results from Islandviewer and Alien_hunter (data not shown). Likewise, it is reported that *S. Typhimurium*, since it diverged from its last common ancestor with *E*. *coli*, has acquired and retained more than 200 discrete regions and these regions even cover more than one-quarter of *Salmonella*'s total genetic materials [35]. Moreover, many recently acquired foreign genes and ORFans came from a still largely unexplored reservoir rather than originated via transfer from distant cellular sources [2]. These imply that there may be more genomic islands undetected by previous methods. Here, considering that a confident prediction is more valuable for comparison, a cut-off with a TSP density five times higher than the genome background was selected, and the results are shown in Figure 2. The average numbers of GIs detected by Islandviewer, GIST and Alien_hunter are 33.1, 51.1 and 76.7, respectively. It is consistent with the previous evaluation that Islandviewer, the one combining the results of three methods, is regarded as the most precise of the extant methods, and Alien_hunter is more sensitive but with a lower accuracy [29], [36]. To check the details, we compared the results of GIST to those of Islandviewer, and the comparison results are displayed with BRIG [37] (see Dataset S6). The comparison of the genome of *Salmonella* 62:z4, z23: – RSK2980 is presented as an example (see Figure 3). The percentage of consistent results between GIST and Islandviewer is about 74.5%. As shown in Figure 3, three main divergent regions that are detected by GIST but missed by Islandviewer are labeled with green triangles. Notably, by referring to the database VFDB [38], we found that three pathogenicity islands (cluster of virulence factor genes) are embedded in three distinct regions, and they are illustrated as follows. The cluster *bcfABCDEFG* carried in the Gist32 and Gist33 regions, encoding fimbrial adherence determinants, is required for intestinal persistence [39]. The virulence factor cluster *pltAB* coding for toxin and *mgtBC* for magnesium uptaking, and these two clusters are located in Gist28 and Gist43, respectively. These results suggest that many omitted pathogenicity islands can be identified by GIST even with a TSP density of five times higher than the genome background, implying the robustness of GIST in GI detection.

Further, to examine whether GIs detected by GIST favors carrying of the classical features of GIs, in the following sections, we compared the known features of GIs among the three groups: GIs detected by GIST; those predicted by Islandviewer; and the genome as a whole, respectively.

### Comparative Analysis: Distribution of gene function categories

Some subsets of genes with specific functions are more likely to be embedded in GI regions [40], [41]. To examine whether the distributions of gene function categories in GIs detected by GIST also differ from the counterparts of the host genomes and the GIs predicted by other methods, we assigned all the genes in the 10 genomes based on the orthologous groups of proteins (COG) database in which the genes are classified into 22 clusters of functional categories. In addition, genes without COG assignments, such as “hypothetical” genes, are classified into another group named “out_COG”. Similarly, the functions of genes in GIs are also identified (see Materials and Methods, Dataset S9). Then, we compared the corresponding proportion of certain functional categories between the genomes and GI candidates predicted by GIST and Islandviewer, separately. In Table 1, the GIs predicted by GIST carry the functional categories [M], [N], [U], [V], [W], [R], [S] and out_COG with significantly higher percentage (t test, p≤0.05), and with a significantly lower percentage in 12 other categories ([C], [E], [F], [G], [H], [I], [P], [Q], [J], [D], [O] and [T]), respectively. Interestingly, eight out of the 12 categories with a lower proportion ([C], [E], [F], [G], [H], [I], [P], and [Q]) are from the functional cluster “Metabolism”. It suggests that most GIs cover fewer genes related to metabolism, although metabolism islands are also identified [15]–[17]. The underrepresented [J], [D], [O] and [T] ([J] Translation, ribosomal structure, and biogenesis, [D] Cell cycle control, cell division, chromosome part, [O] Post-translational modification, protein turnover and [T] Signal transduction mechanisms) may be explained by the fact that those genes, such as coding for two-component systems (TCSs), are more conserved or universal [42], and thus horizontal transfer is not necessary. It is also consistent with the observation that translation and transcription-related genes are far less likely to be horizontally transferred [43]. Moreover, for categories with a higher proportion, the result is consistent with a previous report that categories [M], [N], [U], and [V] ([M] Cell wall/membrane/envelope biogenesis, [N] Cell motility, [U] Intracellular trafficking, secretion, and vesicular transport, [V] Defense mechanisms) were predominant in GIs, confirming that modification of the cell envelope, cell motility, secretion, and protection of cellular DNA are major issues of GIs [40]. It is also rational that many genes in GIs are more “poorly characterized” ([R], [S], and out_COG) because these genes may be transferred from hitherto unculturable and unstudied organisms [21]. Here, another category [W], Extracellular structures, is found significantly overrepresented in GIs. It is most likely that the organisms examined here are gram-negative bacteria which have an outer membrane structure, and have evolved to include a remarkable variety of secretion systems with structurally and functionally divergence [44].

**Table 1:** Comparison of GIST and Islandviewer on detection of GIs in different function categories.

	Average percentage (%)			*p* value^a^	
Function category	Genome	GIST	Islandviewer	Genome-GIST	Genome-Islandviewer
**Information storage and processing**					
**A: RNA processing and modification**	0.03^b^	0	0	-	-
**J: Translation, ribosomal structure, and biogenesis**	4.13	1.24	2.16	**	-
**K: Transcription**	7.01	6.68	4.58	-	-
**L: Replication, recombination, and repair**	4.08	3.51	4.13	-	-
**Cellular processes and signaling**					
**D: Cell cycle control, cell division, chromosome part**	0.78	0.28	0.32	**	*
**M: Cell wall/membrane/envelope biogenesis**	5.41	7.48	7.48	**	**
**N: Cell motility**	2.65	3.58	2.49	**	-
**O: Posttranslational modification, protein turnover**	3.46	1.07	3.5	**	-
**T: Signal transduction mechanisms**	4.06	2.81	2.29	**	**
**U: Intracellular trafficking, secretion, and vesicula**	3.01	4.38	4.59	**	*
**V: Defense mechanisms**	1.08	1.57	0.9	*	-
**W: Extracellular structures**	0.01	0.14	0	*	*
**Metabolism**					
**C: Energy production and conversion**	6.24	3.37	1.79	**	**
**E: Amino acid transport and metabolism**	7.95	4.32	1.87	**	**
**F: Nucleotide transport and metabolism**	2.02	1.02	0.14	**	**
**G: Carbohydrate transport and metabolism**	8.12	3.41	4.15	**	**
**H: Coenzyme transport and metabolism**	3.91	2	0.27	**	**
**I: Lipid transport and metabolism**	2.05	1.43	0.65	*	**
**P: Inorganic ion transport and metabolism**	4.54	1.63	1.12	**	**
**Q: Secondary metabolites biosynthesis**	1.49	0.28	0.1	**	**
**Poorly Characterized**					
**R: General function prediction, S: Function unknown, Out_COG**	27.98	39.74	47.39	**	**

a: Based on the t test, the * represents the *p*<0.05, the ** denotes the *p*<0.001 and the – indicates p>0.05.

b: The number is the average percentage (%) of corresponding function category in 10 strains.

Furthermore, when compared to the result obtained by Genome-Islandviewer, as indicated in Table 1, similar spectra are also found. The differences are only found in categories [J], [N], [O], and [V] ([J] Translation, ribosomal structure, and biogenesis, [N] Cell motility, [O] Post-translational modification, protein turnover, and [V] Defense mechanisms). Further analysis indicated that, compared to Islandviewer, the GIs predicted by GIST cover functional categories [N] and [V] with a significantly higher percentage (t test, p = 0.01 and p<0.0003, respectively) and [O] with a significantly lower percentage (t test, p<0.0007). Genes in [N] and [V] encode additional functions that are not essential for bacterial growth but provide advantages under particular conditions [45]. Obviously, the skewed proportion of GI genes in [N] and [V], similar to the observation by Merkl [40], is attributed to improvement of bacterial fitness. Furthermore, the proteins or pathways involved in post-translational modification [O] are usually strictly conserved in bacteria [46], [47]. So, the lower ratio of GI genes in functional category [O] seems reasonable. Thus, the results suggest that GIs detected by GIST cover the spectrum of functional categories not only similar to outputs by Islandviewer, but also to the results reported previously. Meanwhile, the difference between the results from GIST and Islandviewer also demonstrates that GIST has a higher sensitivity to detect the regions embracing genes with a GI-like function.

### Comparative Analysis: Divergence of subcellular locations

To inspect whether the genes in GIs detected by GIST have preferential localizations, we predicted the subcellular location of each gene across the 10 genomes by using the software PSORTb version 3.0 [48]. To facilitate analysis, the subcellular location of each GI was also assigned (see Materials and Methods, Dataset S10). Then, we compared the proportion of each category of subcellular location between the genome and the predicted GI. As shown in Table 2, the genes in GIST-predicted GIs belonging to the categories of Extracellular, OuterMembrane and Unknown occupied a significantly higher proportion (*t* test, *p* = 5.14E-07, 8.7E-06 and 5.76E-09, respectively), but showed a significantly lower proportion in the categories of Cytoplasmic and Cytoplasmic Membrane (*t* test, *p* = 2.17E-07 and 8.6E-07, respectively). The assembly of OuterMembrane proteins to shape various secretion systems for exporting Extracellular virulence factors is crucial for virulence [49]. So, it is easy to imagine why the GIs, which implant many virulence-related genes [9], have a higher proportion of genes found in the Extracellular and OuterMembrane categories. As the software PSORTb is based on BLAST to assign respective locations, a great proportion of genes with unknown subcellular location in GIs may be caused by the absence of orthologous matches in the current sequence databases – for example, acquired foreign genes coming from still largely unexplored organisms [2]. Moreover, it is reported that the proportion of integrated phage (a source of horizontal gene transfer (HGT)) proteins is predicted to be lower in the Cytoplasmic Membrane category [21], which may partly account for the observation. The genes in the category of Cytoplasmic mainly play structural roles inside the cell, rather than serving particular functions such as virulence. Hence, the mentioned results are comprehensible, hinting that the genes in GIs detected by GIST have a biased GI-like subcellular localization.

**Table 2:** Comparison of GIST and Islandviewer on detection of GIs in different subcellular locations.

	Average number (Percentage)			*p* value^a^	
Subcellular location	Genome	GIST	Islandviewer	Genome-GIST	Genome-Islandviewer
**Cytoplasmic**	2062.1 (42.60)^b^	128.2 (34.00)	107 (31.94)	**	**
**Cytoplasmic-Membrane**	1152 (23.80)	68.9 (18.00)	56 (16.86)	**	**
**Extracellular**	70.5 (1.40)	15.7 (4.20)	10.6 (3.21)	**	*
**Outer Membrane**	97.5 (2.00)	13.4 (3.50)	8.1 (2.51)	**	-
**Periplasmic**	155.2 (3.20)	12.4 (3.30)	6.1 (1.92)	-	**
**Unknown**	1317.8 (26.70)	141.6 (36.40)	152.5 (43.57)	**	**

a: Based on the t test, the * represents the *p*<0.05, the ** denotes the *p*<0.001 and the – indicates p>0.05.

b: The number in the bracket is the average percentage (%) of corresponding subcellular location in 10 strains.

Further, as shown in Table 2, similar repertoires are demonstrated between GIs detected by GIST and Islandviewer. But, there are still significant differences for the categories “OuterMembrane” and “Periplasmic” among the results predicted by GIST and Islandviewer (*t* test, *p* = 7.74E-05 and 6.78E-04, respectively). This indicates that potential novel GIs from GIST hold significantly higher levels of genes in the OuterMembrane and the Periplasmic not only than the genomes, but also the GIs from Islandviewer. Proteins present in the OuterMembrane, serving as sensors and transporters, are in the foremost position in interactions with the host tissue and the immune system, thus suggesting their vital roles in bacterial adaptation [50]. Also, it is reported that cell-surface-related genes tend to be more prevalent in horizontally acquired regions [41]. Besides, many periplasmic proteins, such as DsbA and members in the flagellar protein family revealed by GIST, contribute to pathogenesis of the organism [51]–[53]. Thus, consistent with our foregoing observation of a remarkably higher ratio of genes in Cell wall/membrane/envelope biogenesis (M) and Extracellular structures (W), it makes biological sense that a higher proportion of genes from GIs predicted by GIST are located in the OuterMembrane and the Periplasm. This further supports the claim that this transcription signal-based method GIST can identify the islands embedded with the genes with a GI-like subcellular location.

### Comparative Analysis: GC property and gene length

In a bacterial genome, a major portion of the sequence (70%–80%) shows homogenous GC contents, and the rest of the sequence with divergent GC property is usually designated as “genomic islands” [54]. Based on this feature, several programs were developed to scan the GIs across genomes [55]. To acquire more insights into whether this typical composition feature is also observed among the GIs predicted by GIST and the genomes used in this study, we calculated the GC contents and lengths of all the genes in the 10 genomes (see Materials and Methods). Firstly, GC property and gene length are compared between the genes in GIs predicted by GIST and the genomes. It is shown that the genes in GIs present significantly lower GC contents and shorter lengths (average: 51.7% and 896 for genome, 45.6% and 842 for GIs; ANOVA: *F* = 3039.62, *p*<0.0001 and *F* = 26.95, *p*<0.0001, respectively). Such lower GC content in the GI regions is in accordance with previous findings [56], [57]. Oliver *et al*. also reported that the long coding genes in prokaryotes are GC-rich, whereas the short ones are GC-poor [58]. Interestingly, the GC content and the gene length are both found to be related to codon usage bias, and all these three factors are shown to influence gene expression [59]–[62]. This partly supports the rationality of covering distinct transcriptional signals in GI genes. And these characters of GIs – significantly lower GC content and shorter length – may be favored during evolution for dexterous expression. Furthermore, the GIs specifically predicted by GIST have a significantly lower GC content than the counterparts predicted by Islandviewer (ANOVA, *F* = 365.21, *p*<0.0001). It suggests that compared to those predicted by Islandviewer, the islands predicted by GIST have the GC property much further away from the genome itself, further implying the potency of our method to detect GIs as “alien” elements to the host genome.

### Hints of “non-optimal” codon usage bias

Based on the fact that GIs are usually acquired from perhaps taxonomically unrelated species through HGT, the feature of atypical codon usage is broadly used by previous methods to identify GIs. Here, whether the genes in GIs detected by GIST also have biased codon usage is further examined (see Materials and Methods). Firstly, we compared the effective number of codons (*Nc* value) among the three mentioned groups. The results show that there is a very significant difference between the genes in GIs detected by GIST and those of the genomes (average *Nc* value: 49.68 and 46.72 for GIST genes and genome, respectively; *p*<1E-16). Similarly, the difference is also significant between genes in GIs predicted by Islandviewer and those of the genomes (average *Nc* value: 50.58 and 46.72 for Islandviewer genes and genome, respectively; *p*<1E-22). But, no significant difference was detected between the genes in GIs predicted by GIST and those by Islandviewer. These suggest that the genes within the predicted GIs, with a significantly higher *Nc* value, carry a lower extent of codon bias. This result, together with the finding that GI genes also have a shorter length, is consistent with a previous report that codon bias and gene length are positively correlated [63]. In addition, it implies that the GI genes detected by GIST, similar to the counterparts by Islandviewer, are undergoing selective pressure to use a smaller subset of codons for integrating into the genome context. To further test this bias, the codon adaptation index (CAI) representing the frequency of “preferred” codons [64], is calculated and compared among the three groups. The results indicate that the CAIs of the genes in GIs predicted by GIST and those by Islandviewer are both significantly lower than that of the genomes (average: 0.314, 0.269 and 0.252 for the genome, GI genes detected by GIST and Islandviewer, respectively; *p*<1E-16 and *p*<1E-25 for the comparisons of genomes *vs* GIST and genome *vs* Islandviewer, respectively). This means that the codons of the GI genes detected by GIST or Islandviewer are less optimal. When the GIs are transferred from one species to another, it is reasonable that the codon usage, which has already adapted to the donor genome, may not be optimal in the recipient context [65]. Moreover, preferred codons are usually recognized by more abundant transfer RNAs (tRNAs) in the genomes [66], [67]. The expression efficiency increases through faster recognition of optimal codons, and, in the meantime, expression accuracy decreases with incomplete translation and translation errors when non-optimal codons are used at a cost of overall fitness [68]–[71]. Consequently, a problem emerges: for the recipient genome, how should it express these “foreign” GI genes, with non-preferred codons, efficiently and accurately? Interestingly, by mimicking the horizontal transfer of an antibiotic resistance gene in *E. coli*, Dolors *et al*. reported that the fitness cost of non-optimal codon usage might be rapidly compensated by evolution in regulatory regions [72]. Thus, our finding of a special transcription signal in GIs, just as a snap of evolution in regulatory regions, probably attributed to the optimal expression of the non-optimal codon usage genes in GIs.

### Application of GIST to analyze GIs in the German *E. coli* O104:H4 outbreak strain

The *E. coli* strain in the recent German outbreak carries the ability to cause haemolytic uraemic syndrome (HUS), which may lead to severe kidney failure and even death. Based on the first sequenced and completed chromosomal genome of strain TY-2482 by the Beijing Genomics Institute (BGI), potential GIs in this strain are detected by our method GIST (see Dataset S11). Compared to two other *E. coli* strains, namely, the enteroaggregative *E. coli* (EAEC) strain 55989 (NC_011748) and the enterohaemorrhagic *E. coli* (EHEC) strain O157:H7 Sakai (NC_002695), their GIs are depicted with BRIG (see Figure S2). The data show that most GIs are conserved among the three strains. These conserved ones are probably the products of more ancient HGT events and contribute little to the divergence of the three examined strains. So, only the non-conserved islands are focused here. As shown in Table 3, 11 islands are conserved only between TY-2482 and EAEC 55989, and three islands (Gist1, Gist11 and Gist58) are TY-2482-specific. Notably, there is no island found conserved between TY-2482 and EHEC Sakai but absent in EAEC 55989. It suggests that, compared to EHEC Sakai, EAEC 55989 is a closer relative of TY-2482. This is consistent with our previous finding by using the alignment-free feature frequency profile (FFP) phylogenetic method during Crowdsourcing analyses [73]. Firstly, within the 11 islands, we found that the Gist15 encodes the *E. coli* O104 antigen gene cluster and mainly contains the operons or replicons *wzXY-NnaACBD* [74]. It confirms the closer relationship between TY-2482 and EAEC strain 55989. Secondly, Gist4 embraces the gene cluster *ycgXEFZ-ymgABC*. Tschowri *et al*. recently reported that, induced by cold or starvation, the *YcgF/YcgE* can activate production of the biofilm matrix as well as acid-resistance genes via the *YmgB/YmgA* and *RcsB* system [75]. It is therefore reasonable to expect an improved survivorship for strains harboring such gene clusters under environmental stress. Another interesting gene cluster is *yhhZ-yrhAB* contained in Gist34, which confers an efficient conversion of methionine to cysteine in *Bacillus subtilis* [76]. Usually, owing to the lack of a bypass from methionine to cysteine in sulfur metabolism, *E. coli* has to use selenomethionine to replace methionine and cannot grow with methionine as the mere sulfur source [77]. Thus, this cluster may contribute to the growth of examined strains on methionine, especially when the sulfur source is limited in the host environment. Moreover, the Gist36 mainly consists of the transposon Tn7. Apart from targeting the replicating DNA, Tn7 also recognizes other forms of DNA damage induced by exposure to UV light, mitomycin C, or phleomycin [78]. By this, the strains harboring Tn7 have the advantage in maintaining chromosomal integrity and in accelerating the genome evolution under UV or antibiotic challenges. Besides, many fimbria or fimbria-like adhesion proteins are also enclosed in the GIs, as shown in Table 3. It has been demonstrated that the existence of the lpf operon coding for long polar fimbriae is important for adherence [79]. Finally, we further examined the three TY-2482-specific islands. Gist11 and Gist58 are possibly derived from Phage cdtI and Tn21, and Gist1 contains a microcin H47 (MccH47) system. A full set of MccH47 genes could be found in Gist1, which is mainly necessary for the microcin production, secretion and the immunity peptide for self-protection against its antibiotic action. Indeed, proteins from the MccH47 system were also previously identified in TY-2482 [80]. The first chromosome-coded MccH47 was found produced by *E. coli* H47 [81]. How did the functional island transfer to the recipient strain TY-2482? Recently, María *et al*. suggested that as a GI in *E. coli*, the MccH47 system probably employs a parasitic strategy for its mobility, and, in this manner, the genes responsible for the site-specific recombination are provided by the recipient bacteria [82]. More interestingly, MccH47 mimics siderophores structurally and through a “Trojan horse” mechanism it interacts with microcins and is recognized as siderophores by outer membrane receptors [83]. So, at least in the environment with iron limitation, the strain carrying MccH47 system has the ability to inhibit the growth of other competitors. Although it is still unknown how big the role of the identified GIs played in the German outbreak, the GIs not only provide a more powerful arsenal especially when the stain TY-2482 confronts environmental stresses such as cold, sulfur or iron limitation and antibiotic, but they also accelerate the divergence from EHEC and EAEC 55989 during evolution. Meanwhile, it further demonstrates the ability of GIST in functional GI detection from a new angle.

**Table 3:** Non-conserved genomic islands among three examined *E. coli* strains.

GIs	Start–End	Operons or replicons	Annotation or Notes
**Gist4** a	161735–169690	*ycgXEFZ-ymgABC*	Activate production of the biofilm matrix [74]
**Gist15** a	1251957–1267105	*wzXY-NnaACBD*	O-antigen gene cluster [73]
**Gist23** a	1903476–1912915	B7LDK9	Integrase; CP4-57 prophage
**Gist27** a	2312266–2319405	-	Putative uncharacterized protein
**Gist28** a	2334883–2342762	D3GU29, B7LG82	Antigen 43 (Ag43) phase-variable biofilm formation autotransporter; CP4-44 prophage
**Gist29** a	2436282–2443184	B7LGI6	Putative fimbrial-like adhesin protein
**Gist34** a	2822740–2830307	*yhhZ-yrhAB*	Conversion of Methionine to Cysteine [75], [76]
**Gist36** a	2894844–2907891	*TnsDCBA*	Tn7-like transposition proteins [77]
**Gist38** a	2982406–2992135	*SfmD, lpfBA*	Long polar fimbria protein [78]
**Gist50** a	3888114–3894384	E1U309, B7LDQ7	Transposase InsAB; Putative Filamentation
**Gist54** a	4186166–4193990	*HtrE*, B7LGK3	Periplasmic pilin chaperone, fimbria-like adhesion
**Gist1** b	775–9220	*mchS3, mchS4, insH*	Microcin H47 system—An *E. coli* Genomic Island [79]–[81]
**Gist11** b	1039464–1046318	N6-adenine-methyltransferase (Phage), *rusA*	Phage cdtI
**Gist58** b	4326970–4337621	B3HAL5	Tn21 resolvase and many uncharacterized proteins

Based on the comparison of non-conserved GIs among the three *E. coli* strains (TY-2482, EAEC strain 55989 and EHEC strain O157:H7),

a denotes islands present only in TY-2482 and EAEC strain 55989 and.

b denotes TY-2482-specific islands. The start–end was defined by the start site of the first gene and the end site of the last gene embraced.

#### The implication of high-density transcription initiation signals in GI regions

To determine whether GC content influences the number of TSPs called, we calculated the Pearson Correlation Coefficients (r value) between the GC content and the number of TSP calls among the 10 examined genomes. The results showed that there is a negative correlation but the correlation is not strong (r^2^ values between 0.435–0.579, *p<*0.0001) (Dataset S13). GC-content was found to influence important features such as the bendability and conformational changes of the DNA double helix, which can in turn affect the interaction (such as binding free energy) between RNAP and promoter DNA to affect transcription [84]–[86]. This property may account partly for the comparatively more TSP calls in the region with lower GC content. Moreover, except the tracts ((T)n- and (A)n(T)n-tracts) with comparatively lower GC content, many other features such as newly found non-coding functional elements and special structure modules are combinatorial utilized to constitute the scoring system of algorithm PlatProm in TSP detection [34]. This may partly explain why the correlation between the number of TSP calls and GC content is not strong. Hence, although the GC-content can influence promoter features and gene expression regulation, the GC property is not enough to reflect the full promoter characteristics. And at least, to orchestrate the expression of “exotic” GIs in host genomes, biased GC property is not the only important component.

Then, why are TSPs enriched in the GI regions compared to other genomic regions? What are the other underlying causes? It is imaginable that the GIs, as alien DNA elements, have to undergo numerous rounds of modification before they are best integrated into the host genome. Here, we propose two reasons from the perspective of evolution (Dataset S12). Firstly, if a GI can stay in its recipient organism, the genes within the GI need to be transcribed at the right time, in the right location and in a coordinated manner with the host genes. Thus, although the foreign DNA segments integrated by the model of “selfish operon” may contain inhouse regulatory genes, they usually recruit the existing transcription factors of the host and embed themselves into the ancestral regulatory system [87], [88]. However, because the GI hardly shares orthologs of transcription factors with the host genome at the time when the transfer event occurs [89], new regulatory relations have to be established after the genes are acquired. New TSPs might emerge by accelerated evolution in regulatory regions. Those newly occurring TSPs allow the GI to recruit suitable transcription factors in the host. A wider spectrum of transcripts for a same group of genes might be produced during the course of evolution, until the time when optimal transcripts are generated. Generally speaking, it will take about 8–22 million years for the transferred genes to be eventually integrated into the host regulatory network [90]. Hence, to eliminate the fitness cost by non-optimal codon usage or absonant transcripts of GI, accelerated evolution in regulatory regions will result in TSPs accumulation in GI region, which are crucial for the eventual integration into host regulatory system. Secondly, according to the “life cycle model” of GIs, the GI region might undergo gene loss (or acquisition) and genetic rearrangements following HGT. And thus the GI is reshaped and reorganized [19]. During this process, former operon was broken and more transcription units were probably formed. Comparatively, in most prokaryotic genomes, more than 70% of genes are organized in operon [91]. Thus, compared to the other regions in genome, the transcription units in the newly reshaped GIs are more scattered before they can be coordinately reorganized into one or several new operons. So, precipitation of TSPs in GIs may exhibit transcriptional incompatibility of the newly reshaped GIs at least.

## Materials and Methods

### Genome sequence and annotation data

The chromosome sequence and annotation of the German *E. coli* O104:H4 outbreak strain TY-2482 were downloaded from GenBank under accession number AFOG01000000. Other sequences of genomes included in this study were retrieved from the NCBI ftp site (ftp://ftp.ncbi.nih.gov/genomes/Bacteria/). Our dataset consisted of nine *Salmonella enterica* strains (*S.* Typhimurium LT2, NC_003197; *S.* Typhi CT18, NC_003198; *S.* Typhi Ty2, NC_004631; *S.* Paratyphi A ATCC 9150, NC_006511; *S.* Choleraesuis SC-B67, NC_006905; *S.* 62:z4,z23: – RSK2980, NC_010067; *S.* Paratyphi B SPB7, NC_010102; *S.* Paratyphi A AKU_12601, NC_011147 and *S.* Paratyphi C RKS4594, NC_012125) and one *E. coli* strain (*E. coli* K-12 MG1655, NC_000913). Only chromosomal sequences, but not plasmid data, were analyzed in this study. Moreover, other corresponding annotation files used in downstream analyses, including “gff”, “fna”, “faa” and “ptt”, were also downloaded from the NCBI ftp site.

### TSP scanning

The PlatProm approach, developed by Ozoline *et al*. [34] to search for potential promoters in the genome through predicting transcription start points with optimal position-specific weight matrices, was adopted to detect the TSPs across all the 10 genomes. For *E. coli* K-12 MG1655 and the German *E. coli* outbreak strain TY-2482, TSPs were extracted by the *E. coli-*specific matrices. Accuracy of the PlatProm approach was previously tested on *E. coli* K-12 MG1655 [34]. Briefly, based on 290 known promoters and two control datasets, 85.5% of the known promoters were correctly recognized by PlatProm (start coordinates of predicted TSPs match with experimentally mapped ones within 2 nucleotides). Here, to obtain more accurate prediction for *Salmonella enterica*, PlatProm was attuned to *S.* Typhimurium LT2 promoters by training compilation with 76 experimentally mapped promoters. Meanwhile, information of 214 TSPs mapped by experimental techniques (primer extension, S1-mapping, 5′-RACE and RNA-seq) was collected (see Table S1). Weight matrices for consensus elements, extended -10 dinucleotides and start positions were improved. Each position on both strands was then scored by the newly equipped PlatProm. Only signals with a cut-off score >7.36 were selected as being reliable (with 4 SD higher than background, p<0.00004). To further test the performance of PlatProm in the genome of *S.* Typhimurium LT2, the known TSPs were compared with their corresponding computational calls and 172 out of 214 (80.37%) TSPs were found correctly detected (Table S1). If we consider TSP calls +/−6 nucleotides also as positive hits, the accuracy of the method could reach 92.06%. These demonstrate the robustness of our method in detecting TSPs, at least in the examined *E. coli* and *Salmonella* genomes. Using this approach, other considered genomes were scanned and the results were attached [see Datasets S1, 2, 3, 4, 5].

### Genomic island dataset construction

Five known methods, namely IslandPick, IslandPath-DIMOB, SIGI-HMM, PredBias and Alien_hunter, were used to detect potential genomic islands [see Figure S1]. The numbers of GIs detected by different methods varied from less than 10 to more than 90 for the same strain. The numbers for IslandPick, IslandPath-DIMOB and SIGI-HMM were less than 40. It is consistent with the evaluation that these three methods have a high accuracy rate and a very low recall rate [36]. To reduce the probability of sampling bias and meanwhile to pay more attention to precision, the results from these three methods were selected. And the results of these methods were combined together by joining the overlap outputs [see Dataset S7]. Recently, they have been integrated into a single web platform Islandviewer (http://www.pathogenomics.sfu.ca/islandviewer/query.php). Further, the putative TSPs were matched to the corresponding GI regions for comparison against the whole genomes.

### GIST: Genomic-island Identification by Signal of Transcription

To assess the heterogeneity of sequence within certain regions, the first problem encountered is the determination of the optimal length for the sliding windows. Here, by splitting the genomes to *n* successive windows, we calculated the *X* value by using different lengths of sliding windows each time (from 500 bp to 4,000 bp with a step of 500 bp). The 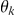 represents the TSP density in the 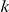 window, and 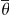 denotes the average density of all the split windows.
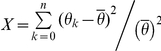



Interestingly, for all the 10 genomes, the first inflexion point of the *X* value emerged when the window length is fixed at 3,000. Thus, we detected all atypical regions with the criteria:

- each window in this region carries the TSP density higher than genome with **y** folds (testing from 1.5 to 6.0 with a step of 0.5); and
- the total successive window must be longer than 4 kb.

Then, to test the optimal number of fold, we calculated the true positive rate (TPR: equivalent to sensitivity) and false positive rate (FPR: equivalent to (1-specificity)) for each fold being tested. To maximize the arithmetic product of sensitivity and specificity, we selected “5 folds” to predict the heterogeneous regions. Finally, based on the GFF file downloaded from NCBI, the genes found within the regions were identified. The boundary of GI was defined by the start site of the first gene and the end site of the last gene embraced. The results are shown in Dataset S8.

### Functional characterization and subcellular localization analysis

The COG assignment results of the 10 genomes were retrieved from the JCVI center (http://www.jcvi.org/). The one-letter code of COG categories is depicted in Table 1. In addition, genes without COG assignments, such as “hypothetical protein”, were classified into another group named “out_COG”. To facilitate the comparison, the genes in GIs predicted both by GIST and Islandviewer were matched to the corresponding functional annotation. Then, with self-written Perl scripts, we calculated the number of genes deposited in each functional category for genomes and GIs [see Dataset S9]. Consequently, PSORTb version 3.0 was used to predict the subcellular location across all the 10 genomes [48]. During the process, we selected the results with a score higher than 7.5 under the option of “gram-negative”. PSORTb was designed to emphasize precision (or specificity) over recall (or sensitivity) and the results were thus more confident. Similarly, after assigning the genes in GIs with the corresponding results, we calculated the number of genes deposited in each type of subcellular location for genomes and GIs with self-written Perl scripts. The results are shown in Dataset S10.

### Codon usage, GC content and gene length

To further delineate the sequence characteristics of different groups, codon usage, GC content and gene length are considered. Here, Codon usage bias was measured by the effective number of codon (*Nc* value), and the possible traits of expression were estimated by the codon adaptation index (CAI), which reflects the extent of bias towards codons known to be favored in highly expressed genes [64]. Both indexes were calculated with the program CodonW 1.4 (written by John Peden, obtained from http://sourceforge.net/projects/codonw/). Further, based on the GFF files obtained from NCBI, we extracted the GC content and the gene length in all the examined genomes. Then, these two features of GI genes were also measured for comparisons. The raw data are not shown here. To examine to what extent the GC property can influence the number of TSP calls, we adapted the sliding window method (4,000 bp with a step of 1000 bp) and then extracted the sequences and calculated the GC content in each window among all the 10 considered genomes by using Perl scripts. Subsequently, corresponding number of TSP calls in each window was also counted according to the foregoing results. Then, based on SAS (statistical analysis system), we calculated the Pearson Correlation Coefficients (r value) between the GC content and the number of TSP calls among the 10 examined genomes. The data are enclosed in Dataset S13.

### Statistical analyses

Various features of different groups were analyzed with the SAS program. Values in different groups were compared using the t test and ANOVA with F test and ranked by Student Newman Keuls test.

## Supporting Information

Figure S1 **Number of GIs detected by different existing methods.** The y-axis represents the number of GIs detected by the methods, and accession numbers of bacterial genomes are shown along the x-axis.
(DOC)

Figure S2 **BRIG diagram showing GIs in German** ***E. coli*** **O104:H4 strain TY-2482 using GIST.** Genomes of EAEC strain 55989 (NC_011748) and EHEC strain O157:H7 Sakai (NC_002695) are added as references.
(DOC)

Table S1 **Comparison of TSPs mapped experimentally and called by PlatProm within the genome of** ***Salmonella*** **Typhimurium LT2.**
(DOC)

Dataset S1 **Putative transcription start positions scored with PlatProm in** ***S.*** **Typhimurium LT2 (NC_003197) and** ***S.*** **Typhi CT18 (NC_003198).**
(XLS)

Dataset S2 **Putative transcription start positions scored with PlatProm in** ***S.*** **Typhi Ty2 (NC_004631) and** ***S.*** **Paratyphi A ATCC 9150 (NC_006511).**
(XLS)

Dataset S3 **Putative transcription start positions scored with PlatProm in** ***S.*** **Choleraesuis SC-B67 (NC_006905) and** ***S.*** **Paratyphi B SPB7 (NC_010102).**
(XLS)

Dataset S4 **Putative transcription start positions scored with PlatProm in** ***S.*** **62:z4,z23:– RSK2980 (NC_010067) and** ***S.*** **Paratyphi A AKU_12601 (NC_011147).**
(XLS)

Dataset S5 **Putative transcription start positions scored with PlatProm in** ***S.*** **Paratyphi C RKS4594 (NC_012125) and** ***E. coli*** **K-12 MG1655 (NC_000913).**
(XLS)

Dataset S6 **BRIG diagrams showing GIs predicted by GIST and Islandviewer in the nine remaining bacterial strains.** GIs predicted with Islandviewer are marked as Island1, Island2, etc.; and those predicted by GIST are denoted with Gist1, Gist2, etc.
(DOC)

Dataset S7 **GI regions detected by Islandviewer in the 10 examined bacterial genomes.**
(XLS)

Dataset S8 **GI regions predicted by GIST in the 10 examined bacterial genomes.**
(XLS)

Dataset S9 **Distribution of GIs in different function categories in genomes, and detected by GIST and Islandviewer.**
(DOC)

Dataset S10 **Distribution of GIs in different subcellular locations in genomes, and detected by GIST and Islandviewer.**
(DOC)

Dataset S11 **GI regions predicted by GIST in the German** ***E. coli*** **O104:H4 outbreak strain TY-2482.**
(XLS)

Dataset S12 **Diagrams showing proposed reasons for enrichments of TSPs in GI regions.**
(PDF)

Dataset S13 **Pearson Correlation Coefficients (r value) between the GC content and the number of TSP calls among the 10 examined genomes.**
(XLS)
