# Supplementary figures and images for: High-Density Transcriptional Initiation Signals Underline Genomic Islands in Bacteria
Source: PLoS One. 2012 Mar 20;7(3):e33759. doi: 10.1371/journal.pone.0033759 (PMC3309015; doi:10.1371/journal.pone.0033759)

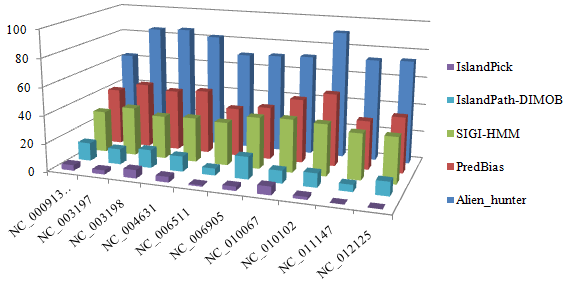

Supplement: Figure S1 — Number of GIs detected by different existing methods. The y-axis represents the number of GIs detected by the methods, and accession numbers of bacterial genomes are shown along the x-axis. (DOC) [file pone.0033759.s001.doc]

**
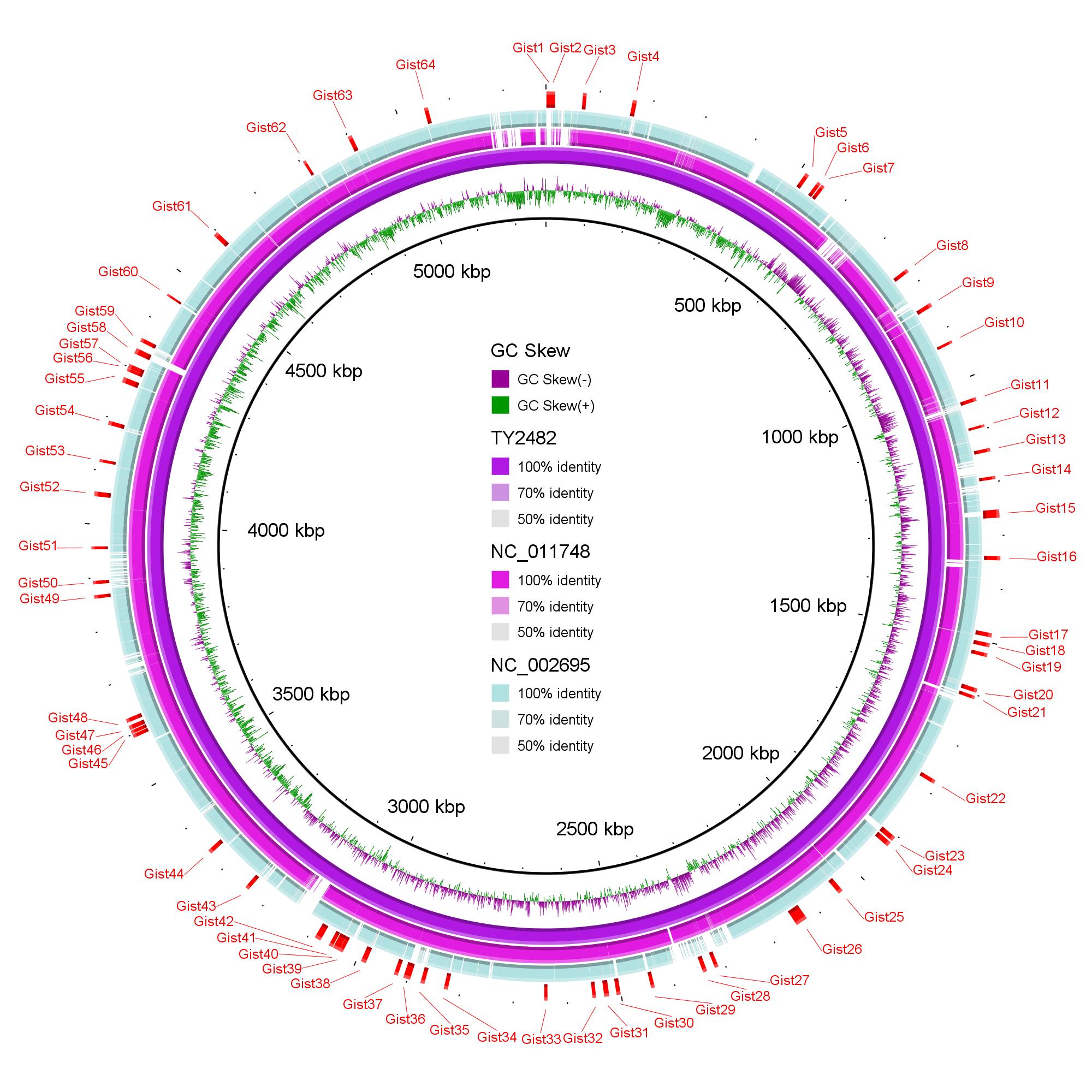
**

Supplement: Figure S2 — BRIG diagram showing GIs in German E. coli O104:H4 strain TY-2482 using GIST. Genomes of EAEC strain 55989 (NC_011748) and EHEC strain O157:H7 Sakai (NC_002695) are added as references. (DOC) [file pone.0033759.s002.doc]

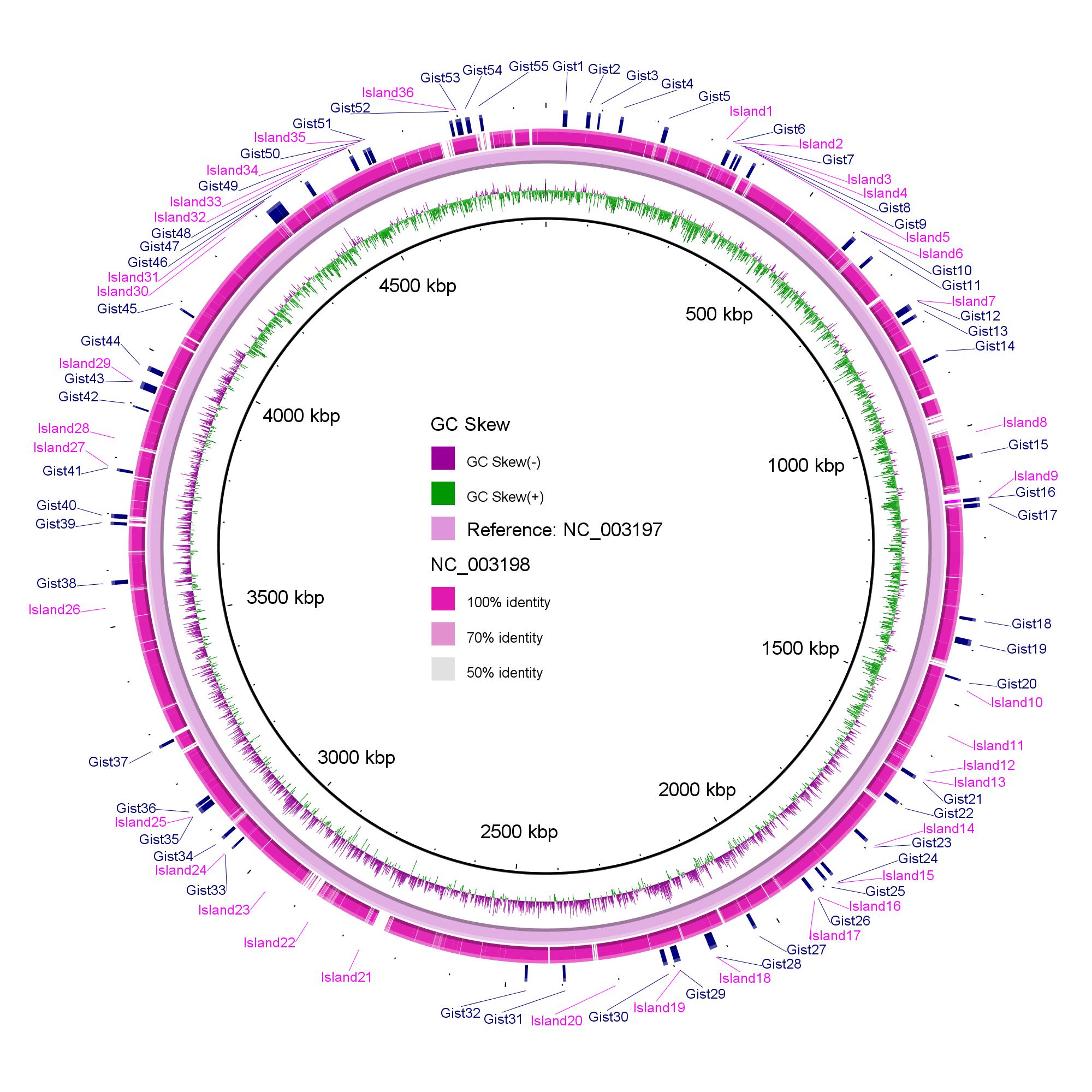


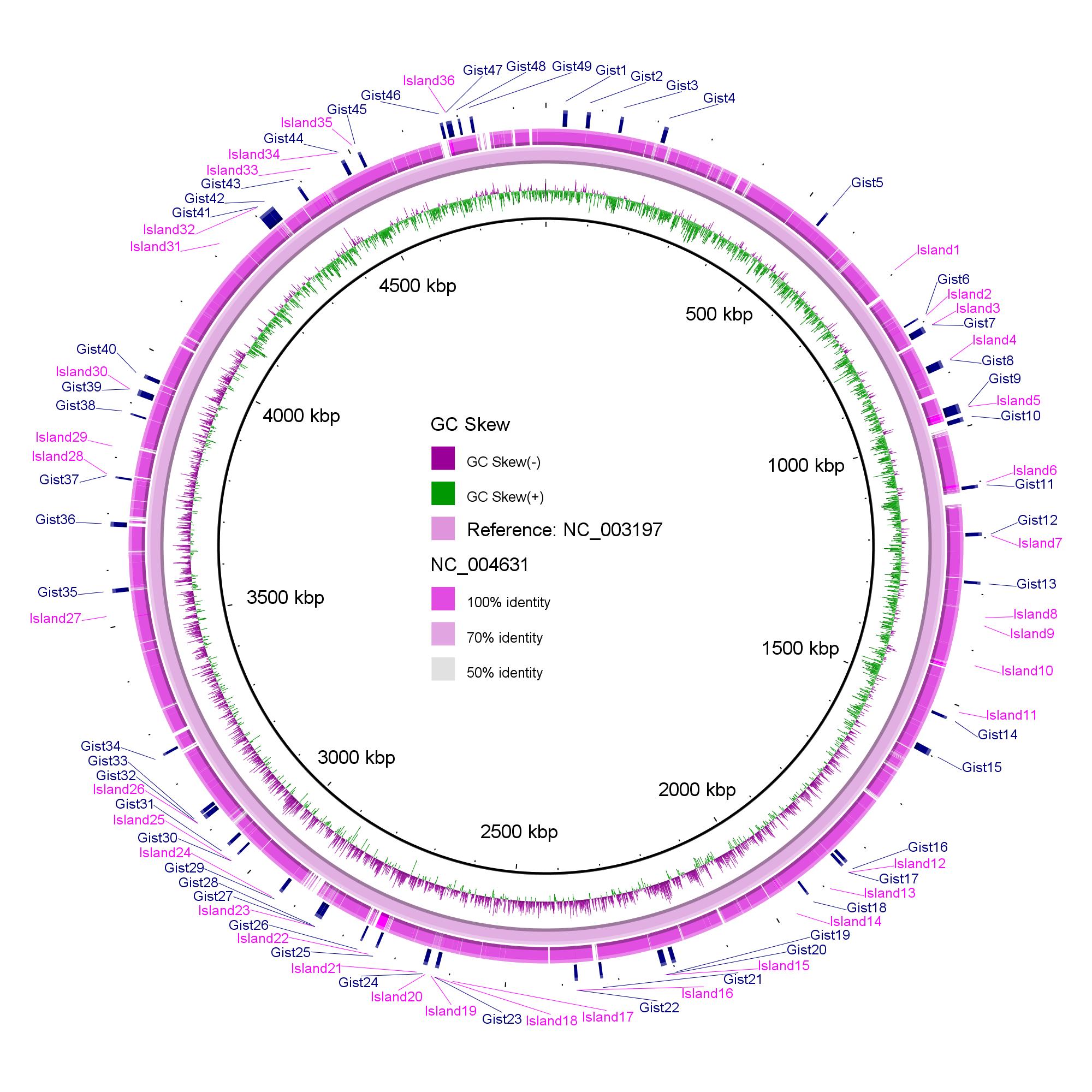


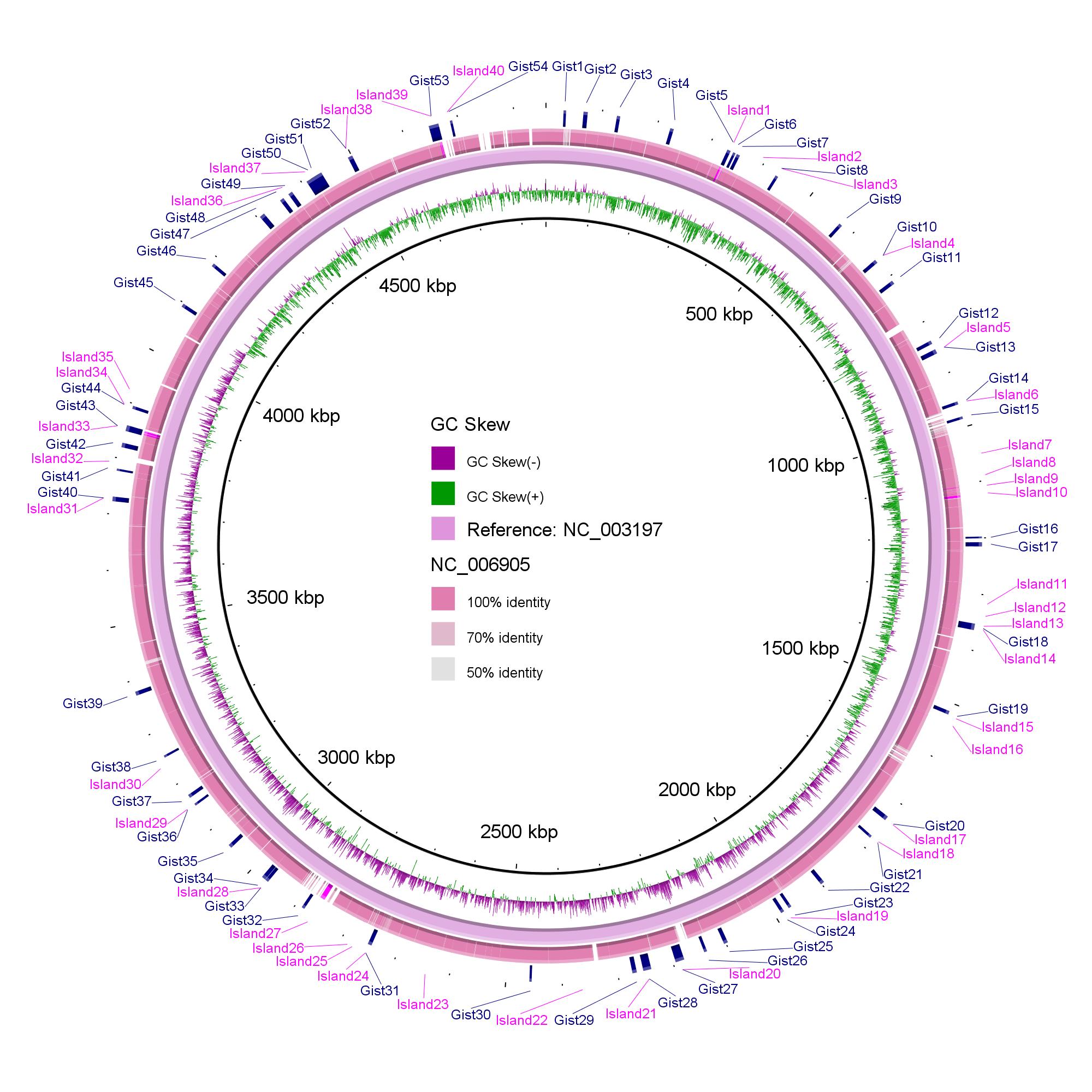


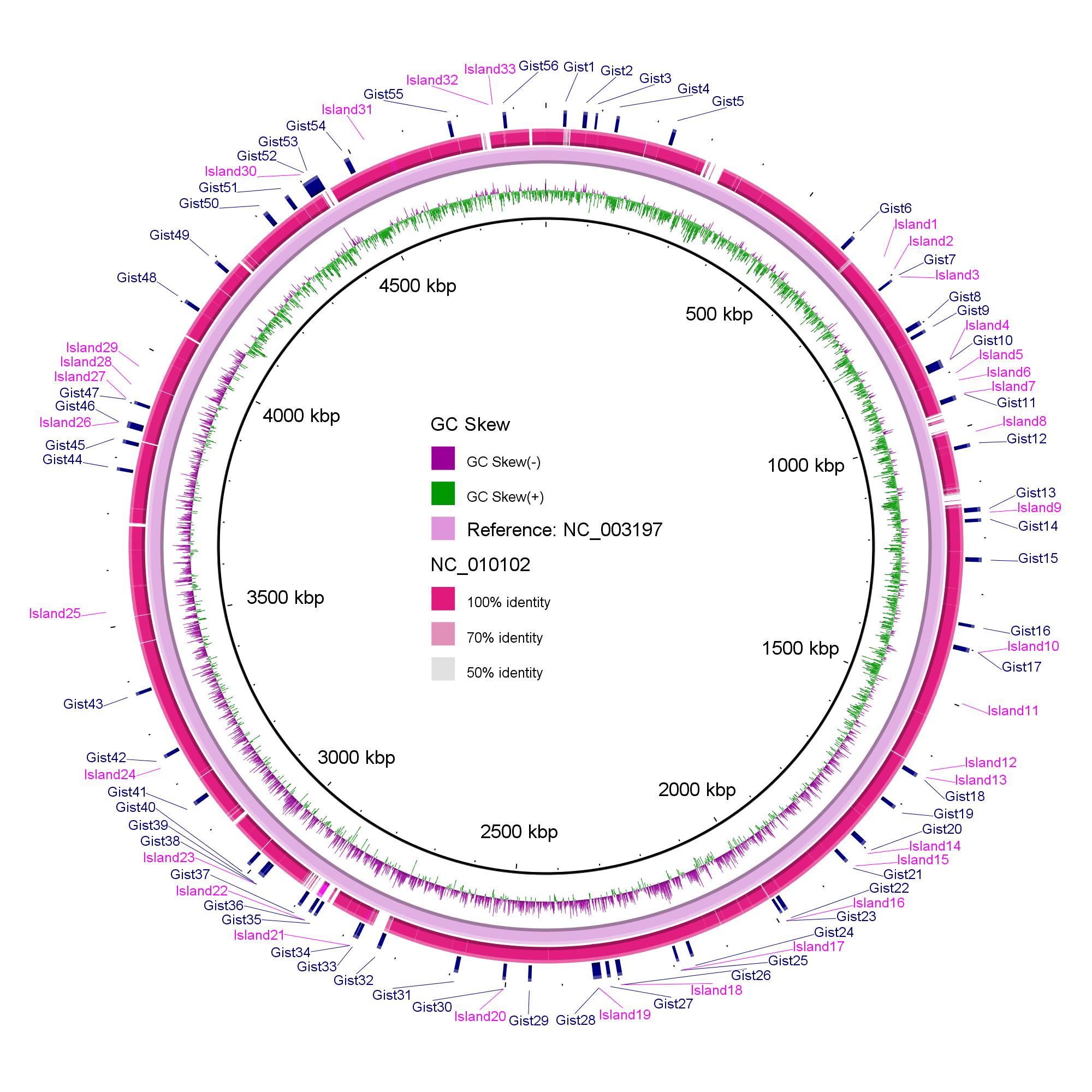


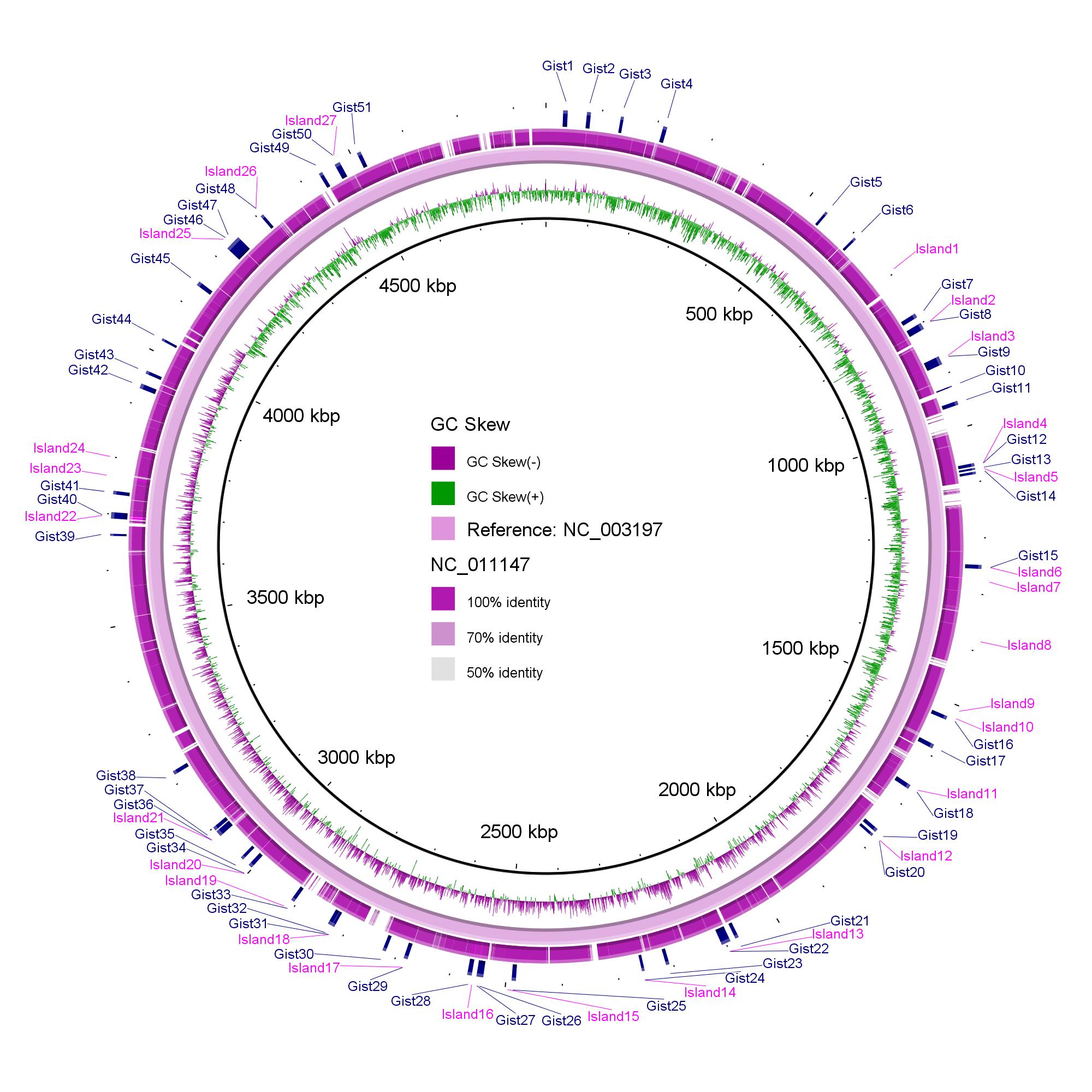


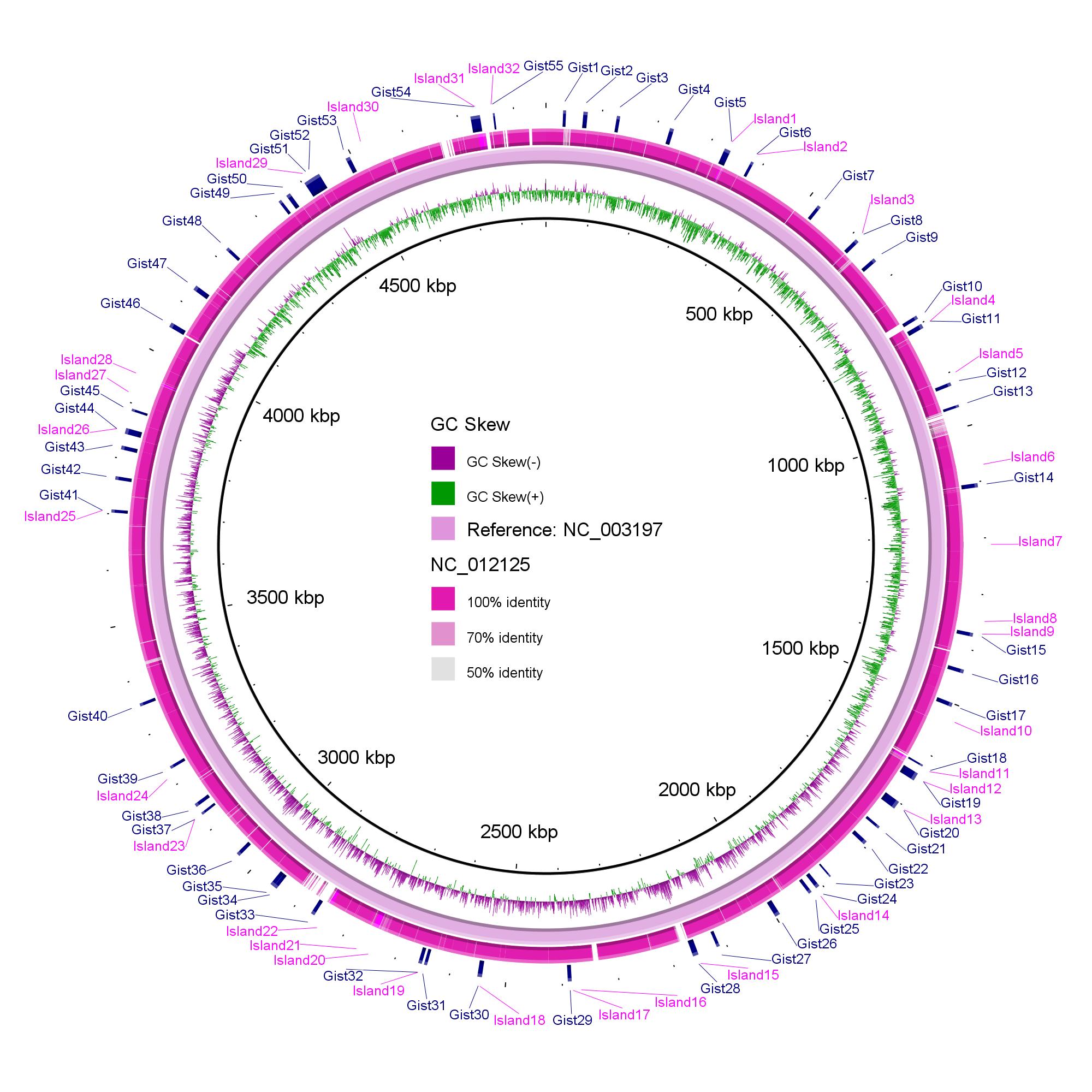


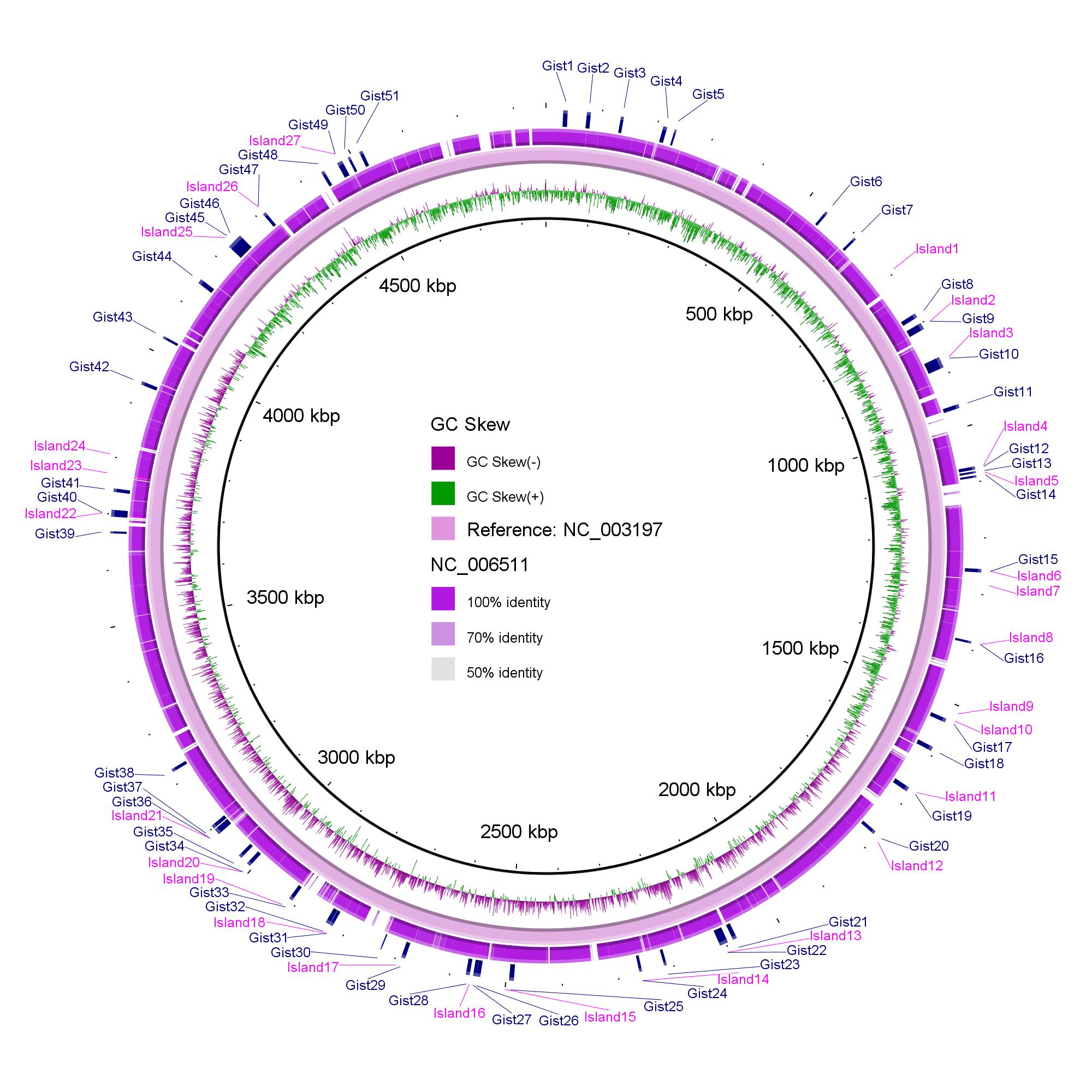


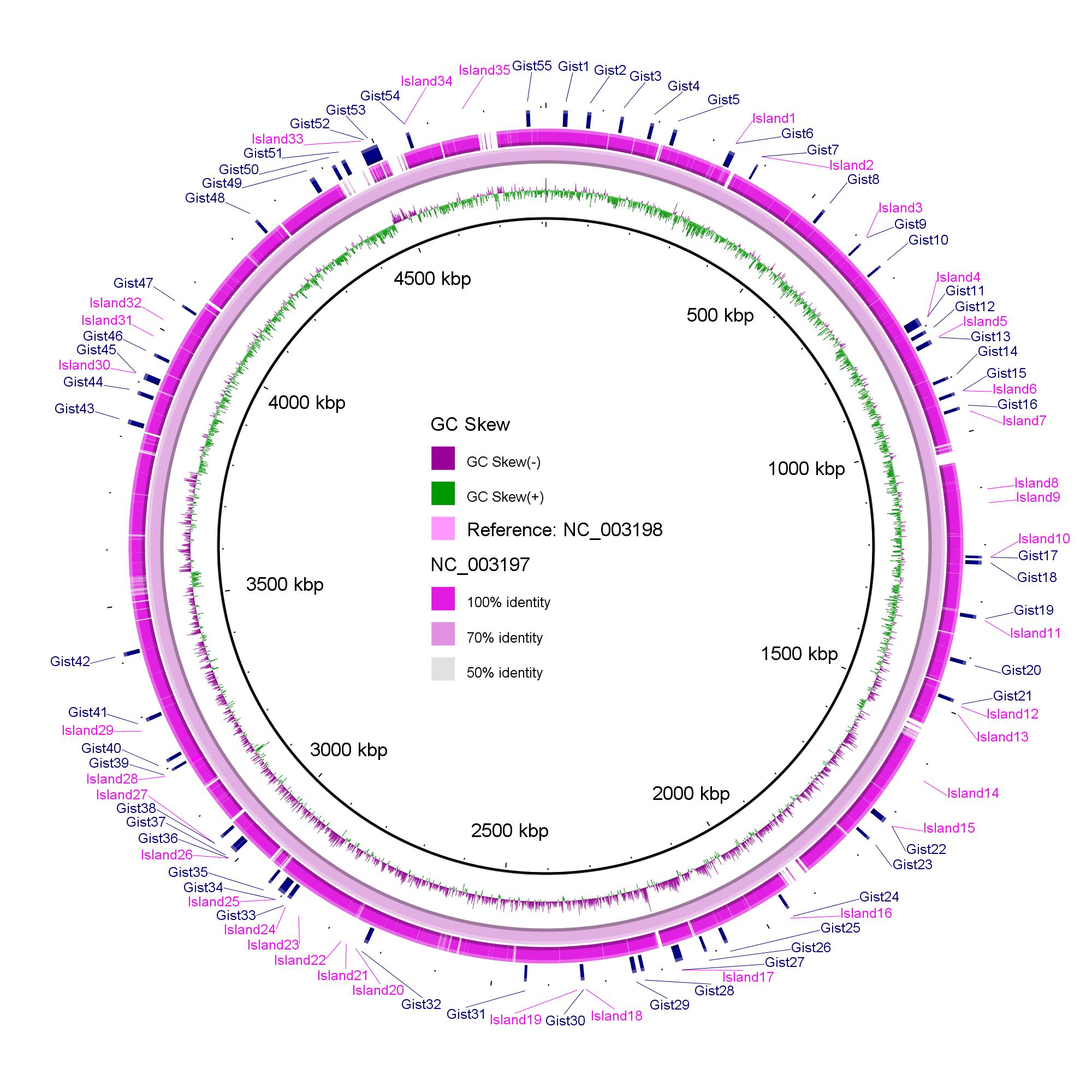


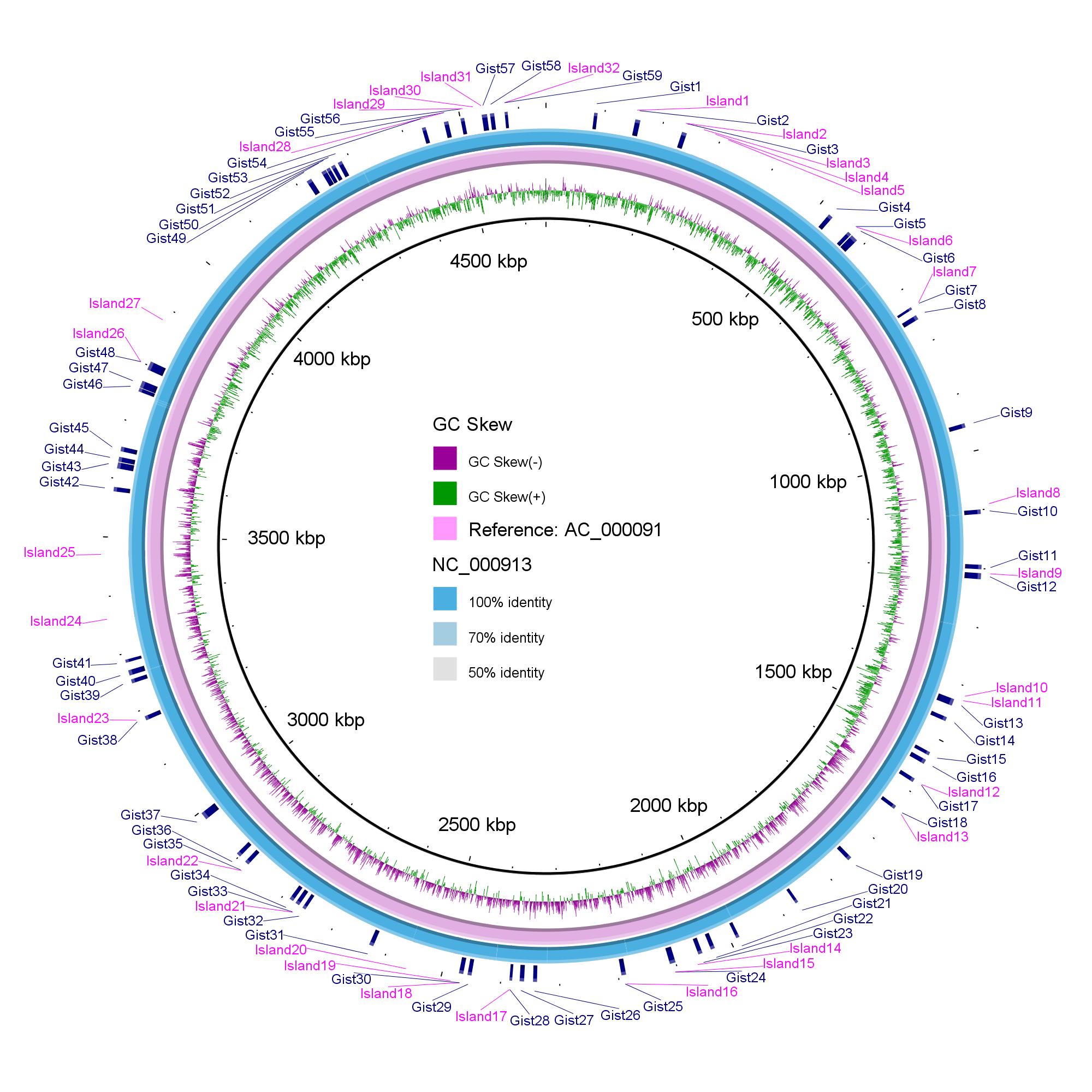

Supplement: Dataset S6 — BRIG diagrams showing GIs predicted by GIST and Islandviewer in the nine remaining bacterial strains. GIs predicted with Islandviewer are marked as Island1, Island2, etc.; and those predicted by GIST are denoted with Gist1, Gist2, etc. (DOC) [file pone.0033759.s009.doc]
