# Supplementary material for: High-Density Transcriptional Initiation Signals Underline Genomic Islands in Bacteria
Source: PLoS One. 2012 Mar 20;7(3):e33759. doi: 10.1371/journal.pone.0033759 (PMC3309015; doi:10.1371/journal.pone.0033759)
Supplement: Table S1 — Comparison of TSPs mapped experimentally and called by PlatProm within the genome of Salmonella Typhimurium LT2. (DOC) [file pone.0033759.s003.doc]

Table S1. Comparison of TSPs mapped experimentally and called by PlatProm**^1^** within the genome of *Salmonella* Typhimurium LT2.

| **Promoter** | **Experiment** | | **PlatProm scan** | | **Promoter** | **Experiment** | | **PlatProm scan** | |
| --- | --- | --- | --- | --- | --- | --- | --- | --- | --- |
| **(strand)**^2^ | **Coordinate(s)** | **Ref.** | **Coordinate**^3^ | **Score** | **(strand)** | **Coordinate(s)** | **Ref.** | **Coordinate** | **Score** |
| **leu(-)** | **133938**^4^ | **1** | **133938** | **7.87** | **ompS-P2(+)** | **2077481, 82** | **59, 60** | **2077482** | **14.81** |
| **panBC-1(-)** | **213296, 97** | **2** | **213290** | **6.92** | **ompS-P1(+)** | **2077511** | **59, 60** | **2077509** | **12.77** |
| **panBC-2(-)*** | **213310** | **2** | **213305** | **11.48** | **cspB(-)** | **2079582** | **61, 4** | **2079580** | **10.54** |
| **htrA-P1(+)** | **244277** | **3** | **244276** | **4.25** | **cob-3/cbiA(-)** | **2114203, 9** | **62, 4** | **2114203** | **6.54** |
| **htrA-P2(+)** | **244445, 46, 52** | **3, 4** | **244441** | **12.45** | **cob-1/cbiA(-)** | **2114232** | **63** | **2114232** | **9.86** |
| **gesA(-)** | **398338** | **5** | **398341** | **6.1** | **cob-P2/cbiA(-)** | **2114263, 75** | **63, 4, 62** | **2114276** | **12.23** |
| **golT(+)** | **398531** | **5** | **398532** | **8.72** | **pocR(-)** | **2115360** | **64** | **2115362** | **6.51** |
| **golB(+)** | **401376** | **5** | **401377** | **7.21** | **pduF-P2(-)** | **2116501** | **64** | **2116498** | **7.13** |
| **iraA/yaiB-1(+)** | **436881** | **6** | **436889** | **8.46** | **pduF-P1(-)** | **2116604** | **64** | **2116608** | **13.12** |
| **iraA/yaiB-2(+)** | **436954** | **6** | **436955** | **10.73** | **pduA(+)** | **2116722** | **64** | **2116722** | **10.57** |
| **brnQ(+)** | **453083, 84, 86** | **116, 4** | **453086** | **12.82** | **hisG(+)*** | **2149387** | **65** | **2149387** | **13.21** |
| **copA(-)** | **560507** | **7** | **560508** | **11.9** | **wzz_st(-)** | **2157849** | **66** | **2157844** | **12.44** |
| **fimZ(-)** | **611121** | **8** | **611106** | **8.24** | **udg/ugd-1(-)*** | **2159244, 47** | **67, 68,4** | **2159245** | **12.89** |
| **fimW(-)*** | **613333** | **9, 4** | **613333** | **15.89** | **udg/ugd-2(-)** | **2159186** | **68** | **2159184** | **10.45** |
| **ramA(+)** | **638853** | **10** | **638850** | **10.05** | **rscDB(+)** | **2367275** | **69, 70** | **2367273** | **7.93** |
| **fepE(+)** | **649550** | **11** | **649551** | **10.91** | **rscB(+)** | **2369939** | **70** | **2369938** | **3.03** |
| **ahpC(+)*** | **670425** | **12, 4** | **670424** | **9.98** | **gyrA(-)** | **2376384** | **71, 4** | **2376387** | **9.59** |
| **pagP(+)** | **692138** | **13** | **692142** | **12.29** | **pmrG(-)*** | **2403776** | **67, 4** | **2403777** | **11.44** |
| **miaB-P1(-)*** | **735947, 48** | **14** | **735947** | **3.77** | **yfbE/pbgP(+)*** | **2403895** | **67, 4** | **2403895** | **12.97** |
| **miaB-P2(-)*** | **735975** | **14** | **735975** | **6.09** | **pmrD(-)*** | **2411601, 2** | **4, 31** | **2411598** | **8.82** |
| **ubiF(+)*** | **736031** | **14** | **736033** | **3.57** | **nuoA-P4(-)** | **2439533** | **72** | **2439531** | **4.43** |
| **dps(-)(s-38)** | **898031** | **15** | **898031** | **9.54** | **nuoA-P3(-)** | **2439583** | **72** | **2439581** | **8.62** |
| **ompX(+)** | **899294** | **13** | **899296** | **10.17** | **nuoA-P2(-)** | **2439626** | **72, 4** | **2439626** | **12.27** |
| **Lrp(+)** | **1036474** | **4** | **1036474** | **9.46** | **nuoA-P1(-)** | **2439803** | **72, 4** | **2439803** | **4.96** |
|  | **1036484** | **16** |  |  | **flk(+)*** | **2483549, 50** | **73** | **2483552** | **4.68** |
| **sodC(-)** | **1130647** | **17** | **1130645** | **7.89** | **pgtP(+)** | **2512086** | **74** | **2512086** | **10.85** |
| **sseI/srfH-1(+)** | **1139779** | **18** | **1139768** | **2.02** | **mntH(-)*** | **2524990** | **75** | **2524989** | **12.87** |
| **sseI/srfH-2(+)** | **1139810** | **18** | **1139807** | **7.61** | **cysK(+)*** | **2543198, 99** | **76, 4** | **2543199** | **9.3** |
| **pyrD(+)*** | **1146929** | **19** | **1146929** | **3.88** | **cysP(-)*** | **2556472** | **77** | **2556472** | **9.3** |
| **pipB-P2(-)** | **1177374, 75** | **20, 4** | **1177375** | **6.76** | **amiA-P1(+)** | **2560226** | **78, 4** | **2560227** | **7.89** |
| **pipB-P1(-)** | **1177508** | **20** | **1177554** | **10.32** | **amiA-P2(+)** | **2560262, 71** | **79** | **2560267** | **3.63** |
|  | **1177554** | **4** |  |  | **shdA(-)** | **2633764** | **46** | **2633765** | **9.08** |
| **sigD/sopB(-)*** | **1179973/4** | **21, 4** | **1179973** | **6.24** | **iscR(-)** | **2683493** | **80** | **2683493** | **12.83** |
| **csgD(-)*** | **1230526** | **22** | **1230526** | **16.36** | **asrA-P1(+)** | **2686374** | **81** | **2686374** | **8.25** |
| **agfB/csgB(+)*** | **1231047, 48** | **23** | **1231048** | **8.78** | **asrA-P2(+)** | **2686386, 88** | **81** | **2686385** | **12.05** |
| **pyrC(-)** | **1249597** | **19, 4** | **1249597** | **3.41** | **glmY(-)** | **2707847** | **82** | **2707847** | **11.91** |
| **flgM(-)** | **1257391** | **24, 4** | **1257391** | **5.32** | **rpoE-P2(-)** | **2780155** | **83** | **2780153** | **6.78** |
| **flgA(-)*** | **1258114/15** | **4, 25** | **1258115** | **14.06** | **rpoE-P1(-)** | **2780220, 22** | **4, 83** | **2780220** | **10.89** |
| **flgB(+)*** | **1258221-24** | **25, 4** | **1258221** | **6.38** | **pipB2(-)** | **2928133** | **46** | **2928132** | **12.99** |
| **flgK(+)** | **1265444** | **26, 4** | **1265432** | **7.83** | **virK(+)** | **2928359** | **46** | **2928350** | **4.65** |
| **cobB-P2(+)** | **1306575** | **27** | **1306572** | **7.91** | **mig-14(+)** | **2929568** | **13** | **2929569** | **10.6** |
| **cobB-P3(+)** | **1306818** | **27** | **1306826** | **9.25** | **nrdH/nrdE(+)** | **2951791** | **84, 85, 4** | **2951790** | **11.41** |
| **sifA(-)** | **1310931** | **28** | **1310943** | **8.03** | **proU/V-P1(+)** | **2955698** | **86, 4** | **2955712** | **6.58** |
| **potA(-)** | **1313232/3** | **4, 29** | **1313233** | **2.9** | **proU/V(+)*** | **2955886-88** | **87** | **2955890** | **8.37** |
| **pepT-P2(+)** | **1313309/11** | **29, 4** | **1313311** | **8.29** | **hilC-P1(-)*** | **3013022** | **88, 4** | **3013022** | **10.23** |
| **pepT-P1(+)** | **1313396** | **29, 4** | **1313396** | **7.74** | **hilC-P2(-)*** | **3013093** | **88** | **3013095** | **8.7** |
| **phoP-P2(-)** | **1319386** | **30, 4** | **1319386** | **7.61** | **orgB(-)** | **3014488** | **89** | **3014489** | **9.86** |
| **phoP-P1(-)*** | **1319397** | **30, 31,4** | **1319396** | **8.21** | **prgH(-)*** | **3017552** | **90, 4** | **3017552** | **14** |
| **msgA-P1(-)** | **1329731** | **4** | **1329731** | **8.66** | **hilD(+)*** | **3017801, 2** | **88, 91,4** | **3017802** | **14.02** |
|  | **1329770** | **32** |  |  | **hilA(+)*** | **3019506, 8** | **92, 4** | **3019508** | **13.14** |
| **msgA-P2(-)** | **1329800** | **4** | **1329800** | **7.82** | **sicA(-)*** | **3031454** | **21, 4** | **3031454** | **10.79** |
| **cspH(-)*** | **1331067** | **33** | **1331067** | **10.83** | **invF-P1(-)** | **3044054** | **93** | **3044056** | **8.35** |
| **pagD(-)*** | **1331478** | **31, 4** | **1331477** | **8.35** | **invF-P2(-)*** | **3044063** | **90, 93, 94, 4** | **3044063** | **8.7** |
| **pagC(+)** | **1331681** | **4** | **1331681** | **14.41** | **invF-P3(-)** | **3044562** | **94** | **3044560** | **6.99** |
| **pagC(+)*** | **1331692** | **31** | **1331692** | **6.36** | **invF-P4(-)** | **3044574** | **94** | **3044571** | **8.1** |
| **agsA(+)*** | **1335458** | **34, 4** | **1335458** | **8.11** | **rpoS(-)*** | **3067052** | **95, 4** | **3067052** | **11.4** |
| **rfc/wzy(-)*** | **1411458** | **35, 4** | **1411457** | **7.34** | **cysJ-P2(-)** | **3092672, 73** | **96** | **3092675** | **6.79** |
| **nlpC(+)** | **1421993** | **36** | **1421993** | **11.91** | **cysJ-P1(-)** | **3092680, 81** | **4, 96** | **3092680** | **14.01** |
| **ssrB(-)** | **1476955** | **37** | **1476953** | **8.71** | **gcvA(-)** | **3135260** | **97, 4** | **3135261** | **6.23** |
| **ssrA(-)** | **1479765, 66** | **37, 4** | **1479766** | **11.01** | **gcvB(+)** | **3135317** | **97** | **3135317** | **11.43** |
| **ssaB(+)** | **1479999** | **38, 4** | **1479992** | **6.16** | **amiC(-)** | **3142829** | **79** | **3142814** | **7.73** |
| **sseA(+)** | **1483281** | **4** | **1483281** | **10.08** | **STM3123(-)** | **3283748** | **46** | **3283747** | **7.3** |
|  | **1483551, 2, 7, 8** | **38** | **1483566** | **6.68** | **STM3124(+)** | **3284036** | **46** | **3284038** | **12.5** |
| **ssaM(+)** | **1493281** | **38** | **1493280** | **7.99** | **metC(+)** | **3320965** | **98** | **3320967** | **6.33** |
| **ssaG(+)** | **1489502** | **38, 4** | **1489502** | **9.89** | **preA/QseB(+)** | **3340374, 75** | **99** | **3340362** | **7.66** |
| **slyA-P3(+)*** | **1519635** | **39** | **1519636** | **3.02** | **ygiW(-)** | **3340679** | **4** | **3340680** | **10.19** |
| **slyA-P2(+)*** | **1519688** | **39** | **1519686** | **5.67** | **preA/QseB(+)** | **3340771** | **4** | **3340771** | **7.82** |
| **slyA-P1(+)*** | **1519844** | **39, 40,4** | **1519844** | **12.78** | **ygiW(-)** | **3340807** | **99** | **3340811** | **4.93** |
| **slyB(-)*** | **1520935** | **31, 4** | **1520935** | **10.59** | **tdcA(-)** | **3413440, 41** | **4, 100** | **3413440** | **11.02** |
| **ompR(-)*** | **1550872** | **41** | **1550872** | **14.9** | **yhdG(+)** | **3555237, 39** | **101, 4** | **3555238** | **9.65** |
| **rstA(-)*** | **1552041** | **13** | **1552040** | **7.39** | **ppiA(-)** | **3621870** | **46** | **3621870** | **10.69** |
| **dcp(+)*** | **1589665** | **42, 4** | **1589663** | **7.05** | **yhiV-P1(-)** | **3774408** | **102** | **3774416** | **7.98** |
| **marR(-)*** | **1597959, 60** | **43, 4** | **1597958** | **11.35** | **yhiV-P2(-)** | **3774538** | **4** | **3774542** | **5.73** |
| **aac/nmpC(+)*** | **1655103, 4** | **44** | **1655104** | **10.7** | **dctA(-)** | **3791609** | **103, 4** | **3791609** | **11.87** |
| **pdgL/pcgL(-)*** | **1690769, 70** | **4, 31** | **1690769** | **12.56** | **amgR(+)** | **3964345** | **104** | **3964334** | **5** |
| **ugtL(-)** | **1691815** | **4** | **1691815** | **8.53** | **mgtC(-)*** | **3965466, 67** | **4, 31** | **3965465** | **12.72** |
| **ugtL(-)*** | **1691820** | **45** | **1691820** | **6.22** | **deoK-P2(-)** | **3994544** | **105** | **3994544** | **9.32** |
| **sifB-P1(+)** | **1692071** | **46** | **1692071** | **5.96** | **deoK-P1(-)*** | **3994549, 50** | **4, 105** | **3994550** | **11.02** |
| **sifB-P2(+)** | **1692127-29** | **28** | **1692128** | **4.81** | **deoQ(+)*** | **3994764** | **105, 4** | **3994764** | **19.24** |
| **sseJ(+)** | **1721244** | **28** | **1721249** | **15.91** | **glmZ(+)** | **4141647** | **82** | **4141635** | **4.48** |
| **STM1632(-)** | **1723350** | **46** | **1723346** | **11.14** | **cyaA-P1(+)*** | **4146014** | **106** | **4146015** | **6.17** |
| **STM1633(+)** | **1723563** | **46** | **1723562** | **10.6** | **cyaA-P2(+)*** | **4146221** | **106** | **4146221** | **12.46** |
| **ogt(+)** | **1753592, 3** | **47, 4** | **1753592** | **3.33** | **metR(-)** | **4169551, 2, 4** | **107, 4** | **4169552** | **9.37** |
| **pyrF(-)** | **1801375, 78** | **4, 48** | **1801375** | **5.56** | **metE(+)** | **4169580,2,3, 9** | **107,4,98** | **4169581** | **6.76** |
| **cysB-P1(-)*** | **1809450** | **49** | **1809450** | **10.38** | **udp(+)** | **4174821, 3, 4** | **108, 4** | **4174821** | **3.07** |
| **cysB-P2(-)** | **1809475** | **50** | **1809475** | **8.28** | **dsbA-P1(+)** | **4204108** | **109** | **4204098** | **3.21** |
| **ompW(-)** | **1828560** | **51, 4** | **1828560** | **11.52** | **dsbA-P2(+)** | **4204165** | **109** | **4204150** | **4.43** |
| **hemA-P1(-)*** | **1875748, 49** | **52, 4,53** | **1875747** | **11.03** | **hemN(+)*** | **4211069** | **110, 4** | **4211069** | **9.49** |
| **hemA -P2(-)** | **1875839-41** | **4, 52,53** | **1875844** | **7.99** | **metJ-P2(-)*** | **4309508, 14** | **111, 112** | **4309509** | **7.65** |
| **lolB(+)** | **1875886** | **4** | **1875886** | **6.07** | **metJ-P1(-)*** | **4309573** | **111, 112** | **4309573** | **13.79** |
| **mgrB(-)*** | **1937087** | **31, 4** | **1937087** | **10** | **metB(+)*** | **4309676-8** | **111, 112** | **4309679** | **14.49** |
| **sopE2(-)** | **1952757** | **4** | **1952757** | **6.2** | **metF(+)** | **4318462,3,5, 6** | **98** | **4318462** | **11.42** |
| **pagM(-)*** | **1962338** | **31, 4** | **1962339** | **4.832** | **metA(+)** | **4400793** | **113** | **4400795** | **3.61** |
| **pagK(+)*** | **1963124, 25** | **4, 31** | **1963125** | **11.92** | **metH(+)** | **4408309** | **114** | **4408310** | **5.27** |
| **flhB(-)*** | **2011457, 58** | **25, 4** | **2011458** | **5.69** | **siiA(+)** | **4477387** | **115, 4** | **4477390** | **10.69** |
| **flhD-P1(-)*** | **2022270** | **54** | **2022270** | **9.62** | **pmrC/yjdB(-)** | **4536050** | **67, 4** | **4536045** | **9.85** |
| **flhD-P3(-)** | **2022363** | **54** | **2022363** | **7.09** | **rtsA(-)** | **4561917** | **117** | **4561918** | **13.96** |
| **flhD-P4(-)** | **2022423, 25** | **4, 54** | **2022425** | **9.61** | **phoN(-)*** | **4564373** | **31, 4** | **4564373** | **11.1** |
| **flhD-P5(-)** | **2022597, 98** | **54, 4** | **2022598** | **8.58** | **mgtA-P1(+)*** | **4699423, 4** | **4, 118** | **4699423** | **11.67** |
| **sdiA(-)** | **2040406** | **55** | **2040405** | **12.47** | **mgtA-P2+)** | **4699467** | **118** | **4699467** | **11.06** |
| **fliA(-)*** | **2045495** | **56, 25,4** | **2045496** | **5.7** | **argI(-)** | **4712789** | **119** | **4712787** | **8.49** |
| **fliD(+)*** | **2049348, 50, 51** | **4, 25,57** | **2049348** | **12.45** | **miaE(+)** | **4712886** | **119** | **4712888** | **3.96** |
| **fliE(-)*** | **2056345, 47** | **25, 4** | **2056347** | **12.01** | **hilE-P3(+)** | **4763220, 23** | **120, 4** | **4763220** | **6.89** |
| **fliL(+)*** | **2063030, 31** | **4, 25** | **2063029** | **5.88** | **hilE-P2'(+)** | **4763395, 9** | **120, 4** | **4763398** | **13.33** |
| **asRNA-fliR(-)** | **2067196/7** | **58** | **2067194** | **6.51** | **hilE-P2(+)*** | **4763408** | **121** | **4763410** | **13.34** |
| **rcsA(+)** | **2067295** | **58** | **2067295** | **11.83** | **hilE-P1(+)*** | **4763501** | **121** | **4763510** | **6.67** |

**^1^**The computation of the σ^70^-specific position weight matrices for the promoters of *Salmonella* was performed by the method of successive iterations (expectation maximization) exactly as it was described previously (<http://www.matbio.org/2011/Ozoline2011(6_t1).pdf>). Promoters used for training are marked with asterisks.

**^2^**The table is mainly composed of σ^70^ TSPs; however, the presence of promoters recognized by alternative σ-factors cannot be excluded. There is also a possibility that some experimentally mapped 5’-ends belong to the processed RNAs.

**^3^**Coordinates corresponding to the positions with maximum score in the cluster covering experimentally mapped TSPs. In the absence of expected cluster, we indicated position with maximum score within ±20 bp region.

**^4^**If the coordinates of the predicted TSPs match with the experimentally mapped ones (+/- 2 nucleotides), the TSPs are considered as correctly identified and highlighted in green; if the predicted TSPs are located in neighboring region of the experimentally mapped ones within +/- 6 nucleotides, they are highlighted in blue.

## References

1. Gemmill, R. M., J. Jones, G. Haughn, and J. M Calvo. 1983, Transcription initiation sites of the leucine operons of Salmonella typhimurium and Escherichia coli. J. Mol. Biol. 170:39-59.
2. Aileen Rubio and D. M. Downs 2002 Elevated Levels of Ketopantoate Hydroxymethyltransferase (PanB)Lead to a Physiologically Significant Coenzyme A Elevation in Salmonella enterica Serovar Typhimurium, J. Bacteriol, 184 (10), p. 2827–2832.
3. Lewis, C., Skovierova, H., Rowley, G., Rezuchova, B., Homerova, D., Stevenson, A., Spencer, J., Farn, J., Kormanec, J. and Roberts, M. (2009) Salmonella enterica Serovar Typhimurium HtrA: regulation of expression and role of the chaperone and protease activities during infection. Microbiology, 155, 873-881.
4. Vinoy K Ramachandran1, Neil Shearer1, Jobin J Jacob, Cynthia M Sharma, and Arthur Thompson (2012) The architecture and ppGpp-dependent expression of the primary transcriptome of *Salmonella* Typhimurium during invasion gene expression. *BMC Genomics* 2012, 13:25 doi:10.1186/1471-2164-13-25.
5. Lucas B. Pontel, Maria E. Perez Audero, Martin Espariz, Susana K. Checa and Fernando C. Soncini (2007) GolS controls the response to gold by the hierarchical induction of *Salmonella*-specific genes that include a CBA efflux-coding operonMolecular Microbiology (2007) 66(3), 814–825.
6. Xuanlin Tu, Tammy Latifi, Alexandre Bougdour, Susan Gottesman, and Eduardo A. Groisman (2006) The PhoP_PhoQ two-component system stabilizes the alternative sigma factor RpoS in Salmonella enterica PNAS, vol. 103 (36) 13503–13508.
7. Espariz, M., Checa, S.K., Audero, M.E.P., Pontel, L.B. and Soncini, F.C. (2007) Dissecting the Salmonella response to copper. *Microbiology*, 153, 2989-2997.
8. Kirsty A. McFarland, Sacha Lucchini, Jay C. D. Hinton, and Charles J. Dorman, The Leucine-Responsive Regulatory Protein, Lrp, Activates Transcription of the *fim* Operon in *Salmonella enterica* Serovar Typhimurium via the *fimZ* Regulatory Gene (2008) J. Bacteriol., 190 (2), p. 602–612.
9. Tinker, J.K., Hancox, L.S. and Clegg, S. (2001) FimW Is a Negative Regulator Affecting Type 1 Fimbrial Expression in *Salmonella enterica* Serovar Typhimurium. *J. Bacteriol.*, 183, 435-442
10. S. Baucheron, F. Coste, S. Canepa, M.-C. Maurel, E. Giraud, F. Culard, B. Castaing, A.Roussel and A.Cloeckaert (2012) Binding of the RamR Repressor to Wild-Type and Mutated Promoters of the *ramA* Gene Involved in Efflux-Mediated Multidrug Resistance in *Salmonella enterica* Serovar Typhimurium. *Antimicrob. Agents Chemother.* 2012, 56(2):942.
11. M. M. Pescaretti, F. E. López, R. D. Morero, and Mó. A. Delgado. (2011) **The PmrA/PmrB regulatory system controls the expression of the *wzz_fepE_* gene involved in the O-antigen synthesis of *Salmonella enterica* serovar Typhimurium.** **Microbiology,** 157: 2515 - 2521.
12. Tartaglia, L.A., Storz, G. and Ames, B.N. (1989) Identification and molecular analysis of oxyR-regulated promoters important for the bacterial adaptation to oxidative stress *Journal of Molecular Biology* 210, 709-719.
13. J. C. Perez and E. A. Groisman (2009) Transcription factor function and promoter architecture govern the evolution of bacterial regulons. PNAS, 2009, 106(11) 4319–4324.
14. B. Esberg, H.-C. E. Leung, H.-C. T. Tsui, G. R. Bjork, and M. E. Winkler (1999) Identification of the *miaB* Gene, Involved in Methylthiolation of Isopentenylated A37 Derivatives in the tRNA of *Salmonella typhimurium* and *Escherichia coli.* J. Bacteriol., 181(23), p. 7256–7265.
15. Y. A. Young, S. W. Kim, J. E. Yu, Y. H. KIM, J. Cha, J.-I. Oh, S. K. Eo, J. H. Lee, and H. Y. Kang (2007) Requirement of Fur for the Full Induction of dps Expression in Salmonella enterica Serovar Typhimurium. J. Microbiol. Biotechnol., 17(9), 1452–1459.
16. McFarland, K.A. and Dorman, C.J. (2008) Autoregulated expression of the gene coding for the leucine-responsive protein, Lrp, a global regulator in *Salmonella enterica* serovar Typhimurium. *Microbiology*, 154, 2008-2016.
17. Ye. A. Golubeva and J. M. Slauch (2006) ***Salmonella enterica* Serovar Typhimurium Periplasmic Superoxide Dismutase SodCI Is a Member of the PhoPQ Regulon and Is Induced in Macrophages**. **J. Bacteriol.,** 188(22), 7853 - 7861.
18. X. Feng, D.Walthers, R. Oropeza and L. J. Kenney (2004) The response regulator SsrB activates transcription and binds to a region overlapping OmpR binding sites at *Salmonella* pathogenicity island 2 Molecular Microbiology 54(3), 823–835.
19. Sorensen, K.I., Baker, K.E., Kelln, R.A. and Neuhard, J. (1993) Nucleotide pool-sensitive selection of the transcriptional start site in vivo at the *Salmonella typhimurium* pyrC and pyrD promoters. *J. Bacteriol.*, 175, 4137-4144.
20. Knodler, L.A., Celli, J., Hardt, W.-D., Vallance, B.A., Yip, C. and Finlay, B.B. (2002) Salmonella effectors within a single pathogenicity island are differentially expressed and translocated by separate type III secretion systems. *Molecular Microbiology*, 43, 1089-1103.
21. Darwin, K.H. and Miller, V.L. (2001) Type III secretion chaperone-dependent regulation: activation of virulence genes by SicA and InvF in *Salmonella typhimurium*. *EMBO J*, 20, 1850-1862.
22. U. Romling, Z. Bian, M. Hammar, W. D. Sierralta, and S. Normark (1998) Curli Fibers Are Highly Conserved between *Salmonella typhimurium* and *Escherichia coli* with Respect to Operon Structure and Regulation. J. Bacteriol, 180(3), p. 722–731.
23. S.K. Collinson, S.C. Clouthier, J.L. Doran, P.A. Banser, and W.W. Kay (1996)
    ***Salmonella enteritidis* agfBAC operon encoding thin, aggregative fimbriae**
    **J. Bacteriol.,** 178: 662 - 667.
24. Gillen, K.L. and Hughes, K.T. (1991) Molecular characterization of flgM, a gene encoding a negative regulator of flagellin synthesis in *Salmonella typhimurium*. *J. Bacteriol.*, 173, 6453-6459.
25. Ikebe, T., Iyoda, S. and Kutsukake, K. (1999) Promoter analysis of the class 2 flagellar operons of Salmonella. *Genes & Genetic Systems*, 74, 179-183.
26. Kutsukake, K. and Ide, N. (1995) Transcriptional analysis of the *flgK* and *fliD* operons of *Salmonella typhimurium* which encode flagellar hook-associated proteins. *Molecular and General Genetics MGG*, 247, 275-281.
27. A. C. Tucker and J. C. Escalante-Semerena (2010) Biologically Active Isoforms of CobB Sirtuin Deacetylase in *Salmonella enterica* and *Erwinia amylovora* J. Bacteriol, 192(23), p. 6200–6208.
28. D. Walthers, Y. Li, Y. Liu, G. Anand, J. Yan, and L. J. Kenney (2011) *Salmonella enterica* Response Regulator SsrB Relieves H-NS Silencing by Displacing H-NS Bound in Polymerization Mode and Directly Activates Transcription J.Biol.Chem., 286(3) 1895-1902.
29. Lombardo, M.J., Lee, A.A., Knox, T.M. and Miller, C.G. (1997) Regulation of the *Salmonella typhimurium* pepT gene by cyclic AMP receptor protein (CRP) and FNR acting at a hybrid CRP-FNR site. *J. Bacteriol.*, 179, 1909-1917.
30. Soncini, F.C., Vescovi, E.G. and Groisman, E.A. (1995) Transcriptional autoregulation of the *Salmonella typhimurium* phoPQ operon. *J. Bacteriol.*, 177, 4364-4371.
31. Lejona, S., Aguirre, A., Cabeza, M.L., Vescovi, E.G. and Soncini, F.C. (2003) Molecular Characterization of the Mg2+-Responsive PhoP-PhoQ Regulon in *Salmonella enterica*. *J. Bacteriol.*, 185, 6287-6294.
32. Gunn, J.S., Alpuche-Aranda, C.M., Loomis, W.P., Belden, W.J. and Miller, S.I. (1995) Characterization of the *Salmonella typhimurium* pagC/pagD chromosomal region. *J. Bacteriol.*, 177, 5040-5047.
33. B. H. Kim, I. S. Bang, S. Y. Lee, S. K. Hong, S. H. Bang, I. S. Lee and Y. K. Park (2001) Expression of *cspH*, Encoding the Cold Shock Protein in *Salmonella enterica* Serovar Typhimurium UK-1 *J. Bacteriol.,* 183(19), 5580.
34. Waldminghaus, T., Heidrich, N., Brantl, S. and Narberhaus, F. (2007) FourU: a novel type of RNA thermometer in Salmonella. *Molecular Microbiology*, 65, 413-424.
35. Wong, D.K.H., Morris, C., Lam, T.L., Wong, W.K.R. and Hackett, J. (1999) Identification of O-antigen polymerase transcription and translation start signals and visualization of the protein in *Salmonella enterica* serovar Typhimurium. *Microbiology*, 145, 2443-2451.
36. T. Wada, Y. Tanabe, and K. Kutsukake (2011) FliZ Acts as a Repressor of the *ydiV* Gene, Which Encodes an Anti-FlhD4C2 Factor of the Flagellar Regulon in *Salmonella enterica* Serovar Typhimurium. J. Bacteriol, 193(19), p. 5191–5198.
37. Feng X., Oropeza R., Kenney L.J. (2003) Dual regulation by phospho-OmpR of
    *ssrA/B* gene expression in *Salmonella* pathogenicity island 2. Mol. Microbiol., 48, p. 1131–1143.
38. Walthers, D., Carroll, R.K., Navarre, W.W., Libby, S.J., Fang, F.C. and Kenney, L.J. (2007) The response regulator SsrB activates expression of diverse Salmonella pathogenicity island 2 promoters and counters silencing by the nucleoid-associated protein H-NS. *Molecular Microbiology*, 65, 477-493.
39. V. A. Norte, M. R. Stapleton, and J. Green (2003), PhoP-Responsive Expression of the *Salmonella enterica* Serovar Typhimurium *slyA* Gene. J. Bacteriol, 185(12) p. 3508–3514.
40. Stapleton, M.R., Norte, V.A., Read, R.C. and Green, J. (2002) Interaction of the *Salmonella typhimurium*Transcription and Virulence Factor SlyA with Target DNA and Identification of Members of the SlyA Regulon. *Journal of Biological Chemistry*, 277, 17630-17637.
41. Marcos Ferna.ndez-Mora, Jose. Luis Puente, and Edmundo Calva (2004) OmpR and LeuO Positively Regulate the *Salmonella enterica* Serovar Typhi *ompS2* Porin Gene J.Bacteriol., 186(10), 2909–2920.
42. Hamilton, S. and Miller, C.G. (1992) Cloning and nucleotide sequence of the *Salmonella typhimurium* dcp gene encoding dipeptidyl carboxypeptidase. *J. Bacteriol.*, 174, 1626-1630.
43. Sulavik, M.C., Dazer, M. and Miller, P.F. (1997) The *Salmonella typhimurium* mar locus: molecular and genetic analyses and assessment of its role in virulence. *J. Bacteriol.*, 179, 1857-1866.
44. S. Magnet, P. Courvalin, and T. Lambert (1999) Activation of the Cryptic *aac(6*9*)-Iy* Aminoglycoside Resistance Gene of *Salmonella* by a Chromosomal Deletion Generating a Transcriptional Fusion. J.Bacteriol, 181(21) p. 6650–6655.
45. Shi, Y., Latifi, T., Cromie, M.J. and Groisman, E.A. (2004) Transcriptional Control of the Antimicrobial Peptide Resistance ugtL Gene by the Salmonella PhoP and SlyA Regulatory Proteins. *Journal of Biological Chemistry*, 279, 38618-38625.
46. G. Zhao, N. Weatherspoon, W. Kong, R. Curtiss, III, and Y. Shi **(2008) A dual-signal regulatory circuit activates transcription of a set of divergent operons in *Salmonella typhimurium*** **PNAS,** 105(52), 20924 - 20929.
47. Ibanez-Ruiz, M., Robbe-Saule, V., Hermant, D., Labrude, S. and Norel, F. (2000) Identification of RpoS (sigma S)-Regulated Genes in *Salmonella enterica* Serovar Typhimurium. *J. Bacteriol.*, 182, 5749-5756.
48. Theisen, M., Kelln, R.A. and Neuhard, J. (1987) Cloning and characterization of the pyrF operon of *Salmonella typhimurium*. *European Journal of Biochemistry*, 164, 613-619.
49. J. Ostrowski, G. Jagura-Burdzy, and N. M. Kredich (1987), DNA Sequences of the *cysB* Regions of *Salmonella typhimurium* and *Escherichia coli* J. Biol. Chem., 262(13), 5999-6005.
50. Ostrowski, J. and Kredich, N.M. (1991) Negative autoregulation of cysB in *Salmonella typhimurium*: in vitro interactions of CysB protein with the cysB promoter. *J. Bacteriol.*, 173, 2212-2218.
51. Gil, F., Hernandez-Lucas, I., Polanco, R., Pacheco, N., Collao, B., Villarreal, J.M., Nardocci, G., Calva, E. and Saavedra, C.P. (2009) SoxS regulates the expression of the *Salmonella enterica* serovar Typhimurium ompW gene. *Microbiology*, 155, 2490-2497.
52. Choi, P., Wang, L., Archer, C.D. and Elliott, T. (1996) Transcription of the glutamyl-tRNA reductase (hemA) gene in *Salmonella typhimurium* and *Escherichia coli*: role of the *hemA* P1 promoter and the *arcA* gene product. *J. Bacteriol.*, 178, 638-646.
53. Elgrably-Weiss, M., Park, S., Schlosser-Silverman, E., Rosenshine, I., Imlay, J. and Altuvia, S. (2002) A *Salmonella enterica* serovar Typhimurium hemA Mutant Is Highly Susceptible to Oxidative DNA Damage. *J. Bacteriol.*, 184, 3774-3784.
54. Yanagihara, S., Iyoda, S., Ohnishi, K., Iino, T. and Kutsukake, K. (1999) Structure and transcriptional control of the flagellar master operon of *Salmonella typhimurium*. *Genes & Genetic Systems*, 74, 105-111.
55. A. L. Turnbull, W. Kim, and M. G. Surette (2012), Transcriptional regulation of sdiA by cAMPreceptor protein, LeuO, and environmental signals in Salmonella enterica serovar Typhimurium. Can. J. Microbiol. 58: 10–22 (2012).
56. Ikebe, T., Iyoda, S. and Kutsukake, K. (1999) Structure and expression of the fliA operon of *Salmonella typhimurium*. *Microbiology*, 145, 1389-1396.
57. Kutsukake, K. and Ide, N. (1995) Transcriptional analysis of the *flgK* and *fliD* operons of *Salmonella typhimurium* which encode flagellar hook-associated proteins. *Molecular and General Genetics MGG*, 247, 275-281.
58. Q. Wang and R. M. Harshey (2009) Rcs signalling-activated transcription of rcsA induces strong anti-sense transcription of upstream fliPQR flagellar genes from a weak intergenic promoter: regulatory roles for the anti-sense transcript in virulence and motility. Molecular Microbiology, 74(1), 71–84.
59. M. A. Flores-Valdez, J. L. Puente and E. Calva (2003) Negative Osmoregulation of the *Salmonella ompS1* Porin Gene Independently of OmpR in an *hns* Background. J. Bacteriol., 185(22), 6497-6506.
60. M. A. De la Cruz, M. Fernandez-Mora, C. Guadarrama, M. A. Flores-Valdez, V. H. Bustamante, A. Vazquez and E. Calva (2007) LeuO antagonizes H-NS and StpA-dependent repression in *Salmonella enterica ompS1.* Molecular Microbiology 66(3), 727–743.
61. Craig, J.E., Boyle, D., Francis, K.P. and Gallagher, M.P. (1998) Expression of the cold-shock gene *cspB* in *Salmonella typhimurium* occurs below a threshold temperature. *Microbiology*, 144, 697-704.
62. Richter-Dahlfors, A.A. and Andersson, D.I. (1991) Analysis of an anaerobically induced promoter for the cobalamin biosynthetic genes in *Salmonella typhimurium*. *Molecular Microbiology*, 5, 1337-1345.
63. M. R. Rondon and J. C. Escalante-Semerena (1996) In Vitro Analysis of the Interactions between the PocR Regulatory Protein and the Promoter Region of the Cobalamin Biosynthetic (*cob*) Operon of *Salmonella typhimurium* LT2 J. Bacteriol, 178(8), p. 2196–2203.
64. Ping Chen, M. Ailion, T. Bobik, G. Stormo, and J. Roth (1995) Five Promoters Integrate Control of the *cob/pdu* Regulon in *Salmonella typhimurium* J. Bacteriol, 177 (19), p. 5401–5410.
65. D. L. Riggs, R. D. Mueller, H.-S. Kwan, and S. W. Artz (1986) Promoter domain mediates guanosine tetraphosphate activation of the histidine operon. Proc. Natl. Acad. Sci. USA, 83, pp. 9333-9337.
66. M. A. Delgado, C. Mouslim and E. A. Groisman (2006) The PmrA/PmrB and RcsC/YojN/RcsB systems control expression of the *Salmonella* O-antigen chain length determinant Molecular. Microbiology 60(1), 39–50.
67. M. M. S. M. Wosten and E. A. Groisman (1999) Molecular Characterization of the PmrA Regulon. J. Biol. Chem., 274(38), p. 27185–27190.
68. Mouslim, C. and Groisman, E.A. (2003) Control of the Salmonella ugd gene by three two-component regulatory systems. *Molecular Microbiology*, 47, 335-344.
69. M. M. Pescaretti, F. E. Lopez, R. D. Morero and M. A. Delgado (2010) Transcriptional autoregulation of the RcsCDB phosphorelay system in Salmonella enterica serovar Typhimurium. Microbiology, 156, 3513–3521.
70. M. M. Pescaretti, R. Morero and M. A. Delgado (2009), Identification of new promoter for the response regulator rcsB expression in Salmonella enterica serovar Typhimurium. FEMS Microbiol Lett, 300, 165–173.
71. Keane, O.M. and Dorman, C.J. (2003) The *gyr* genes of *Salmonella enterica* serovar Typhimurium are repressed by the factor for inversion stimulation, Fis. *Molecular Genetics and Genomics*, 270, 56-65.
72. Archer, C.D. and Elliott, T. (1995) Transcriptional control of the nuo operon which encodes the energy- conserving NADH dehydrogenase of *Salmonella typhimurium*. *J. Bacteriol.*, 177, 2335-2342.
73. J. E. Karlinsey, A. J. Pease, M. E. Winkler, J. L. Bailey and K. T. Hughes (1997) The *flk* Gene of *Salmonella typhimurium* Couples Flagellar Pand L-Ring Assembly to Flagellar Morphogenesis *J. Bacteriol.*, 179(7), 2389-2400.
74. D. Goldrick, G.-Q. Yu, S.-Q. Jiang, and J.-S. Hong (1988) Nucleotide Sequence and Transcription Start Point of the Phosphoglycerate Transporter Gene of Salmonella typhimurium J. Bacteriol, 170(8), 3421-3426.
75. D. G. Kehres, A. Janakiraman, J. M. Slauch, and M. E. Maguire1 (2002) Regulation of *Salmonella enterica* Serovar Typhimurium *mntH* Transcription by H2O2, Fe2, and Mn2 J. Bacteriol, 184(12), p. 3151–3158.
76. Byrne, C.R., Monroe, R.S., Ward, K.A. and Kredich, N.M. (1988) DNA sequences of the *cysK* regions of *Salmonella typhimurium* and *Escherichia coli* and linkage of the *cysK* regions to *ptsH*. *J. Bacteriol.*, 170, 3150-3157.
77. M. M. Hryniewicz and N. M. Kredich (1991), The cysP Promoter of Salmonella typhimurium: Characterization of Two Binding Sites for CysB Protein, Studies of In Vivo Transcription Initiation, and Demonstration of the Anti-Inducer Effects of Thiosulfate. J.Bacteriol., 173(18), p. 5876-5886.
78. Xu, K. and Elliott, T. (1993) An oxygen-dependent coproporphyrinogen oxidase encoded by the hemF gene of *Salmonella typhimurium*. *J. Bacteriol.*, 175, 4990-4999.
79. N. Weatherspoon-Griffin, G. Zhao, W. Kong, Y. Kong, Morigen, H. Andrews-Polymenis, M. McClelland, and Y. Shi (2011) The CpxR/CpxA Two-component System Up-regulates Two Tat-dependent Peptidoglycan Amidases to Confer Bacterial Resistance to Antimicrobial Peptide. J. Biol. Chem. 286(7), p. 5529–5539.
80. J. A. Lewis, J. M. Boyd, D. M. Downs, and J. C. Escalante-Semerena (2009) Involvement of the Cra Global Regulatory Protein in the Expression of the *iscRSUA* Operon, Revealed during Studies of Tricarballylate Catabolism in *Salmonella enterica* J.Bacteriol. 191(7) p. 2069–2076.
81. C. J. Huang. and E. L. Barrett (1991) Sequence Analysis and Expression of the Salmonella typhimurium asr Operon Encoding Production of Hydrogen Sulfide from Sulfite J. Bacteriol., 173(4), 1544-1553.
82. Y. Gopel1, D. Luttmann, A. K. Heroven, B. Reichenbach1, P. Dersch and B. Gorke1 (2011), Common and divergent features in transcriptional control of the homologous small RNAs GlmY and GlmZ in Enterobacteriaceae Nucleic Acids Research, 39(4), 1294–1309.
83. Miticka, H., Rowley, G., Rezuchova, B., Homerova, D., Humphreys, S., Farn, J., Roberts, M. and Kormanec, J. (2003) Transcriptional analysis of the rpoE gene encoding extracytoplasmic stress response sigma factor σE in *Salmonella enterica* serovar Typhimurium. *FEMS Microbiology Letters*, 226, 307-314.
84. A. Panosa, I. Roca, I. Gibert (2010) Ribonucleotide Reductases of Salmonella Typhimurium: Transcriptional Regulation and Differential Role in Pathogenesis. PLoS ONE, 5(6), e11328.
85. Jordan, A., Aragall, E., Gibert, I. and Barbé, J. (1996) Promoter identification and expression analysis of *Salmonella typhimurium* and *Escherichia coli* nrdEF operons encoding one of two class I ribonucleotide reductases present in both bacteria. *Molecular Microbiology*, 19, 777-790.
86. Rajkumari, K., Ishihama, A. and Gowrishankar, J. (1997) Evidence for transcription attenuation rendering cryptic a sigmaS- dependent promoter of the osmotically regulated proU operon of *Salmonella typhimurium*. *J. Bacteriol.*, 179, 7169-7173.
87. D. G. Overdier, E. R. Olson, B. D. Erickson, M. M. Ederer, and L. N. Csonkal (1989) Nucleotide Sequence of the Transcriptional Control Region of the Osmotically Regulated proU Operon of Salmonella typhimurium and Identification of the 5' Endpoint of the proU mRNA J.Bacteriol. 171(9), 4694-4706.
88. Olekhnovich, I.N. and Kadner, R.J. (2002) DNA-Binding Activities of the HilC and HilD Virulence Regulatory Proteins of *Salmonella enterica* Serovar Typhimurium. *J. Bacteriol.*, 184, 4148-4160.
89. A. Aguirre, M. L. Cabeza, S. V. Spinelli, M. McClelland, E. G. Vescovi, and F. C. Soncini (2006) PhoP-Induced Genes within *Salmonella* Pathogenicity Island. J.Bacteriol. 188(19) 6889–6898.
90. Lostroh, C.P. and Lee, C.A. (2001) The HilA Box and Sequences outside It Determine the Magnitude of HilA-Dependent Activation of PprgH from Salmonella Pathogenicity Island 1. *J. Bacteriol.*, 183, 4876-4885.
91. Schechter, L.M., Jain, S., Akbar, S. and Lee, C.A. (2003) The Small Nucleoid-Binding Proteins H-NS, HU, and Fis Affect hilA Expression in *Salmonella enterica* Serovar Typhimurium. *Infect. Immun.*, 71, 5432-5435.
92. Schechter, L.M., Damrauer, S.M. and Lee, C.A. (1999) Two AraC/XylS family members can independently counteract the effect of repressing sequences upstream of the hilA promoter. *Molecular Microbiology*, 32, 629-642.
93. C. P. Lostroh, V. Bajaj and C. A. Lee (2000) The cis requirements for transcriptional activation by HilA, a virulence determinant encoded on SPI-1. Molecular Microbiology, 37(2), 300-315.
94. S. Akbar, L. M. Schechter, C. P. Lostroh and C. A. Lee (2003) AraC/XylS family members, HilD and HilC, directly activate virulence gene expression independently of HilA in *Salmonella typhimurium* Molecular Microbiology. 47(3), 715–728.
95. M. Hirsch and T. Elliott (2005) Fis Regulates Transcriptional Induction of RpoS in *Salmonella enterica* J. Bacteriol., 187(5), 1568–1580.
96. Ostrowski, J. and Kredich, N.M. (1989) Molecular characterization of the cysJIH promoters of *Salmonella typhimurium* and *Escherichia coli*: regulation by cysB protein and N-acetyl-L-serine. *J. Bacteriol.*, 171, 130-140.
97. Sharma, C.M., Darfeuille, F., Plantinga, T.H. and Vogel, J.r. (2007) A small RNA regulates multiple ABC transporter mRNAs by targeting C/A-rich elements inside and upstream of ribosome-binding sites. *Genes & Development*, 21, 2804-2817.
98. M. L. Urbanowski, L. S. Plamann, and G. V. Stauffer (1987) Mutations Affecting the Regulation of the metB Gene of Salmonella typhimurium LT2. J. Bacteriol 169(1), 126-130.
99. Merighi, M., Septer, A., Carroll-Portillo, A., Bhatiya, A., Porwollik, S., McClelland, M. and Gunn, J. (2009) Genome-wide analysis of the PreA/PreB (QseB/QseC) regulon of *Salmonella enterica* serovar Typhimurium. *BMC Microbiology*, 9, 42.
100. Kim, M.-j., Lim, S. and Ryu, S. (2008) Molecular Analysis of the *Salmonella typhimurium* tdc Operon Regulation. *Journal of Microbiology and Biotechnology*, 18, 1024-1032.
101. Osuna, R., Lienau, D., Hughes, K.T. and Johnson, R.C. (1995) Sequence, regulation, and functions of fis in *Salmonella typhimurium*. *J. Bacteriol.*, 177, 2021-2032.
102. Zaharik, M.L., Lamb, S.S., Baker, K.E., Krogan, N.J., Neuhard, J. and Kelln, R.A. (2007) Mutations in *yhiT* enable utilization of exogenous pyrimidine intermediates in *Salmonella enterica* serovar Typhimurium. *Microbiology*, 153, 2472-2482.
103. Baker, K.E., Ditullio, K.P., Neuhard, J. and Kelln, R.A. (1996) Utilization of orotate as a pyrimidine source by *Salmonella typhimurium* and *Escherichia coli* requires the dicarboxylate transport protein encoded by dctA. *J. Bacteriol.*, 178, 7099-7105.
104. E.-J. Lee and E. A. Groisman (2010) An antisense RNA that governs the expression kinetics of a multifunctional virulence genemmi. Molecular Microbiology, 76(4), 1020–1033.
105. Christensen, M., Borza, T., Dandanell, G., Gilles, A.-M., Barzu, O., Kelln, R.A. and Neuhard, J. (2003) Regulation of Expression of the 2-Deoxy-D-Ribose Utilization Regulon, deoQKPX, from *Salmonella enterica* serovar *Typhimurium*. *J. Bacteriol.*, 185, 6042-6050.
106. L. K. Thorner, J. P. Fandl and S. W. Artz (1990) Analysis of Sequence Elements Important for Expression and Regulation of the Adenylate Cyclase Gene *(cya)* of *Salmonella typhimurium.* Genetics, 125, 709-717.
107. Plamann, L.S. and Stauffer, G.V. (1987) Nucleotide sequence of the *Salmonella typhimurium* metR gene and the metR-metE control region. *J. Bacteriol.*, 169, 3932-3937.
108. Zolotukhina, M., Ovcharova, I., Eremina, S., Errais Lopes, L. and Mironov, A.S. (2003) Comparison of the structure and regulation of the *udp* gene of *Vibrio cholerae, Yersinia pseudotuberculosis*, *Salmonella typhimurium*, and *Escherichia coli*. *Research in Microbiology*, 154, 510-520.
109. M. Goecke, C. Gallant, P. Suntharalingam, N.L. Martin (2002) Salmonella typhimurium DsbA is growth-phase regulated. FEMS Microbiology Letters 206 (2002) 229-234.
110. Xu, K. and Elliott, T. (1994) Cloning, DNA sequence, and complementation analysis of the *Salmonella typhimurium* hemN gene encoding a putative oxygen-independent coproporphyrinogen III oxidase. *J. Bacteriol.*, 176, 3196-3203.
111. M.L. Urbanowski and G.V. Stauffer **(1985)** Nucleotide sequence and biochemical characterization of the metJ gene from Salmonella typhimurium LT2**.** **Nucl. Acids Res.,** 13(3), 673-685.
112. M. L. Urbanowski and G. V. Stauffer (1986) Autoregulation by Tandem Promoters of the Salmonella typhimurium LT2 metJ Gene 1986, J. Bacteriol. 165(3) p. 740-745.
113. R. Mares, M. L. Urbanowski, and G. V. Stauffer (1992) Regulation of the Salmonella typhimurium metA Gene by the MetR Protein and Homocysteine. J. Bacteriol. 174(2), p. 390-397.
114. K. A. Byerly, M. L. Urbanowski, and G. V. Stauffer (1991) The MetR Binding Site in the Salmonella typhimurium metH Gene: DNA Sequence Constraints on Activation. J.Bacteriol., 173(11), p. 3547-3553.
115. Main-Hester, K.L., Colpitts, K.M., Thomas, G.A., Fang, F.C. and Libby, S.J. (2008) Coordinate Regulation of Salmonella Pathogenicity Island 1 (SPI1) and SPI4 in *Salmonella enterica* Serovar Typhimurium. *Infect. Immun.*, 76, 1024-1035.
116. Ohnishi, K., Hasegawa, A., Matsubara, K., Date, T., Okada, T. and Kiritani, K. (1988) Cloning and nucleotide sequence of the brnQ gene, the structural gene for a membrane-associated component of the LIV-II transport system for branched-chain amino acids in *Salmonella typhimurium*. *The Japanese journal of genetics*, 63, 343-357.
117. I. N. Olekhnovich and R. J. Kadner (2007) Role of Nucleoid-Associated Proteins Hha and H-NS in Expression of *Salmonella enterica* Activators HilD, HilC, and RtsA Required for Cell Invasion. J. Bacteriol., 189(19), p. 6882–6890.
118. J. Barchiesi, M. E. Castelli, F. C. Soncini, and E. G. Véscovi **(2008) mgtA Expression Is Induced by Rob Overexpression and Mediates a Salmonella enterica Resistance Phenotype**. **J. Bacteriol.,** 190: 4951 - 4958.
119. B. C. Persson and G. R. Bjork (1993) Isolation of the Gene (miaE) Encoding the Hydroxylase Involved in the Synthesis of 2-Methylthio-cis-Ribozeatin in tRNA of Salmonella typhimurium and Characterization of Mutants. J. Bacteriol., 175(24), p. 7776-7785.
120. Lim, S., Yun, J., Yoon, H., Park, C., Kim, B., Jeon, B., Kim, D. and Ryu, S. (2007) Mlc regulation of Salmonella pathogenicity island I gene expression via hilE repression. *Nucleic Acids Research*, 35, 1822-1832.
121. M. Aaron Baxter and Bradley D. Jones (2005) The *fimYZ* Genes Regulate *Salmonella enterica* Serovar Typhimurium Invasion in Addition to Type 1 Fimbrial Expression and Bacterial Motility Infection and Immunity, 73(3), p. 1377–1385.
