# Supplementary material for: High-Density Transcriptional Initiation Signals Underline Genomic Islands in Bacteria
Source: PLoS One. 2012 Mar 20;7(3):e33759. doi: 10.1371/journal.pone.0033759 (PMC3309015; doi:10.1371/journal.pone.0033759)
Supplement: Dataset S9 — Distribution of GIs in different function categories in genomes, and detected by GIST and Islandviewer. (DOC) [file pone.0033759.s012.doc]

| **Function category** | **Genome** | | | | | | | | | |
| --- | --- | --- | --- | --- | --- | --- | --- | --- | --- | --- |
|  | **NC_000913** | **NC_003197** | **NC_003198** | **NC_004631** | **NC_006511** | **NC_006905** | **NC_010067** | **NC_010102** | **NC_011147** | **NC_012125** |
| **[A]** | 2  (0.05) **^a^** | 2  (0.05) | 1  (0.02) | 1  (0.02) | 1  (0.03) | 2  (0.04) | 1  (0.02) | 1  (0.02) | 1  (0.03) | 2  (0.05) |
| **[C]** | 287  (6.95) | 292  (6.76) | 262  (5.56) | 257  (6.00) | 264  (6.72) | 276  (6.02) | 260  (6.00) | 293  (5.39) | 264  (6.75) | 276  (6.24) |
| **[D]** | 33  (0.80) | 37  (0.86) | 36  (0.76) | 33  (0.77) | 32  (0.81) | 34  (0.74) | 33  (0.76) | 37  (0.68) | 32  (0.82) | 35  (0.79) |
| **[E]** | 363  (8.79) | 356  (8.24) | 342  (7.26) | 339  (7.91) | 337  (8.58) | 350  (7.64) | 339  (7.83) | 364  (6.70) | 337  (8.62) | 351  (7.93) |
| **[F]** | 97  (2.35) | 89  (2.06) | 89  (1.89) | 86  (2.01) | 87  (2.21) | 87  (1.90) | 86  (1.99) | 89  (1.64) | 87  (2.23) | 86  (1.94) |
| **[G]** | 368  (8.91) | 393  (9.10) | 339  (7.19) | 339  (7.91) | 341  (8.68) | 352  (7.68) | 324  (7.48) | 395  (7.27) | 343  (8.77) | 361  (8.16) |
| **[H]** | 153  (3.71) | 177  (4.10) | 176  (3.74) | 170  (3.97) | 172  (4.38) | 177  (3.86) | 168  (3.88) | 177  (3.26) | 171  (4.37) | 170  (3.84) |
| **[I]** | 100  (2.42) | 94  (2.18) | 85  (1.80) | 84  (1.96) | 86  (2.19) | 90  (1.96) | 89  (2.05) | 95  (1.75) | 83  (2.12) | 91  (2.06) |
| **[J]** | 182  (4.41) | 185  (4.28) | 182  (3.86) | 182  (4.25) | 184  (4.68) | 180  (3.93) | 172  (3.97) | 180  (3.31) | 182  (4.65) | 177  (4.00) |
| **[K]** | 301  (7.29) | 329  (7.61) | 313  (6.64) | 304  (7.09) | 298  (7.58) | 317  (6.92) | 288  (6.65) | 317  (5.84) | 298  (7.62) | 302  (6.83) |
| **[L]** | 200  (4.84) | 167  (3.86) | 227  (4.82) | 182  (4.25) | 150  (3.82) | 201  (4.39) | 175  (4.04) | 162  (2.98) | 152  (3.89) | 171  (3.87) |
| **[M]** | 226  (5.47) | 260  (6.02) | 239  (5.07) | 240  (5.60) | 230  (5.85) | 236  (5.15) | 222  (5.13) | 249  (4.58) | 228  (5.83) | 238  (5.38) |
| **[N]** | 111  (2.69) | 125  (2.89) | 116  (2.46) | 112  (2.61) | 118  (3.00) | 122  (2.66) | 90  (2.08) | 128  (2.36) | 117  (2.99) | 122  (2.76) |
| **[O]** | 138  (3.34) | 161  (3.73) | 160  (3.40) | 154  (3.59) | 150  (3.82) | 148  (3.23) | 146  (3.37) | 154  (2.84) | 147  (3.76) | 156  (3.53) |
| **[P]** | 217  (5.26) | 202  (4.67) | 194  (4.12) | 190  (4.43) | 188  (4.78) | 199  (4.34) | 211  (4.87) | 206  (3.79) | 188  (4.81) | 193  (4.36) |
| **[Q]** | 64  (1.55) | 67  (1.55) | 63  (1.34) | 63  (1.47) | 64  (1.63) | 68  (1.48) | 63  (1.45) | 68  (1.25) | 64  (1.64) | 66  (1.49) |
| **[T]** | 178  (4.31) | 183  (4.24) | 181  (3.84) | 178  (4.15) | 171  (4.35) | 181  (3.95) | 169  (3.90) | 185  (3.41) | 173  (4.42) | 176  (3.98) |
| **[U]** | 124  (3.00) | 142  (3.29) | 133  (2.82) | 128  (2.99) | 132  (3.36) | 136  (2.97) | 109  (2.52) | 144  (2.65) | 131  (3.35) | 140  (3.16) |
| **[V]** | 49  (1.19) | 49  (1.13) | 47  (1.00) | 45  (1.05) | 46  (1.17) | 48  (1.05) | 50  (1.15) | 48  (0.88) | 46  (1.18) | 42  (0.95) |
| **[W]** | 0  (0.00) | 1  (0.02) | 1  (0.02) | 1  (0.02) | 0  (0.00) | 1  (0.02) | 0  (0.00) | 1  (0.02) | 0  (0.00) | 1  (0.02) |
| **[R], [S],**  **out_COG** | 935  (22.65) | 1010  (23.37) | 1526  (32.39) | 1197  (27.93) | 878  (22.35) | 1376  (30.04) | 1336  (30.85) | 2139  (39.38) | 866  (22.15) | 1268  (28.66) |
| **Total** | 4128  (100.00) | 4321  (100.00) | 4712  (100.00) | 4285  (100.00) | 3929  (100.00) | 4581  (100.00) | 4331  (100.00) | 5432  (100.00) | 3910  (100.00) | 4424  (100.00) |

^a^: The number in the bracket is the average percentage of corresponding function type in 10 strains

| **Function category** | **Genomic islands by GIST** | | | | | | | | | |
| --- | --- | --- | --- | --- | --- | --- | --- | --- | --- | --- |
|  | NC_000913 | NC_003197 | NC_003198 | NC_004631 | NC_006511 | NC_006905 | NC_010067 | NC_010102 | NC_011147 | NC_012125 |
| **[A]** | 0  (0.00) ^a^ | 0  (0.00) | 0  (0.00) | 0  (0.00) | 0  (0.00) | 0  (0.00) | 0  (0.00) | 0  (0.00) | 0  (0.00) | 0  (0.00) |
| **[C]** | 16  (3.70) | 20  (5.63) | 10  (2.60) | 10  (3.27) | 11  (3.21) | 18  (4.53) | 11  (2.77) | 22  (4.06) | 11  (3.16) | 19  (4.67) |
| **[D]** | 1  (0.23) | 1  (0.28) | 2  (0.52) | 1  (0.33) | 1  (0.29) | 1  (0.25) | 1  (0.25) | 2  (0.37) | 1  (0.29) | 1  (0.25) |
| **[E]** | 15  (3.47) | 20  (5.63) | 19  (4.94) | 16  (5.23) | 17  (4.96) | 21  (5.29) | 14  (3.53) | 24  (4.43) | 18  (5.17) | 22  (5.41) |
| **[F]** | 7  (1.62) | 4  (1.13) | 3  (0.78) | 4  (1.31) | 6  (1.75) | 3  (0.76) | 4  (1.01) | 3  (0.55) | 6  (1.72) | 3  (0.74) |
| **[G]** | 13  (3.01) | 22  (6.20) | 16  (4.16) | 12  (3.92) | 12  (3.50) | 15  (3.78) | 9  (2.27) | 18  (3.32) | 15  (4.31) | 15  (3.69) |
| **[H]** | 9  (2.08) | 8  (2.25) | 7  (1.82) | 7  (2.29) | 13  (3.79) | 8  (2.02) | 1  (0.25) | 9  (1.66) | 13  (3.74) | 10  (2.46) |
| **[I]** | 7  (1.62) | 9  (2.54) | 9  (2.34) | 4  (1.31) | 5  (1.46) | 7  (1.76) | 3  (0.76) | 8  (1.48) | 6  (1.72) | 4  (0.98) |
| **[J]** | 8  (1.85) | 4  (1.13) | 4  (1.04) | 5  (1.63) | 6  (1.75) | 6  (1.51) | 2  (0.50) | 7  (1.29) | 5  (1.44) | 7  (1.72) |
| **[K]** | 23  (5.32) | 32  (9.01) | 29  (7.53) | 24  (7.84) | 26  (7.58) | 31  (7.81) | 38  (9.57) | 27  (4.98) | 27  (7.76) | 28  (6.88) |
| **[L]** | 29  (6.71) | 13  (3.66) | 17  (4.42) | 13  (4.25) | 11  (3.21) | 11  (2.77) | 14  (3.53) | 13  (2.40) | 13  (3.74) | 18  (4.42) |
| **[M]** | 29  (6.71) | 34  (9.58) | 34  (8.83) | 30  (9.80) | 33  (9.62) | 34  (8.56) | 30  (7.56) | 30  (5.54) | 34  (9.77) | 30  (7.37) |
| **[N]** | 17  (3.94) | 15  (4.23) | 18  (4.68) | 14  (4.58) | 17  (4.96) | 15  (3.78) | 8  (2.02) | 16  (2.95) | 18  (5.17) | 15  (3.69) |
| **[O]** | 2  (0.46) | 4  (1.13) | 6  (1.56) | 4  (1.31) | 6  (1.75) | 5  (1.26) | 4  (1.01) | 4  (0.74) | 5  (1.44) | 5  (1.23) |
| **[P]** | 14  (3.24) | 6  (1.69) | 4  (1.04) | 3  (0.98) | 6  (1.75) | 9  (2.27) | 7  (1.76) | 11  (2.03) | 3  (0.86) | 10  (2.46) |
| **[Q]** | 1  (0.23) | 3  (0.85) | 0  (0.00) | 0  (0.00) | 1  (0.29) | 3  (0.76) | 0  (0.00) | 1  (0.18) | 1  (0.29) | 2  (0.49) |
| **[T]** | 12  (2.78) | 12  (3.38) | 14  (3.64) | 11  (3.59) | 10  (2.92) | 10  (2.52) | 11  (2.77) | 14  (2.58) | 12  (3.45) | 15  (3.69) |
| **[U]** | 18  (4.17) | 18  (5.07) | 20  (5.19) | 17  (5.56) | 19  (5.54) | 18  (4.53) | 20  (5.04) | 19  (3.51) | 20  (5.75) | 18  (4.42) |
| **[V]** | 10  (2.31) | 6  (1.69) | 6  (1.56) | 5  (1.63) | 7  (2.04) | 6  (1.51) | 11  (2.77) | 5  (0.92) | 7  (2.01) | 4  (0.98) |
| **[W]** | 0  (0.00) | 1  (0.28) | 1  (0.26) | 1  (0.33) | 0  (0.00) | 1  (0.25) | 0  (0.00) | 1  (0.18) | 0  (0.00) | 1  (0.25) |
| **[R], [S],**  **out_COG** | 201  (46.53) | 123  (34.65) | 166  (43.12) | 125  (40.85) | 136  (39.65) | 175  (44.08) | 209  (52.64) | 308  (56.83) | 133  (38.22) | 180  (44.23) |
| **Total** | 432  (100) | 355  (100) | 385  (100) | 306  (100) | 343  (100) | 397  (100) | 397  (100) | 542  (100) | 348  (100) | 407  (100) |

^a^: The number in the bracket is the average percentage of corresponding function type in Genome islands by GIST aomong 10 strains.

| **Function category** | **Genomic islands by Islandviewer** | | | | | | | | | |
| --- | --- | --- | --- | --- | --- | --- | --- | --- | --- | --- |
|  | NC_000913 | NC_003197 | NC_003198 | NC_004631 | NC_006511 | NC_006905 | NC_010067 | NC_010102 | NC_011147 | NC_012125 |
| **[A]** | 0  (0.00) ^a^ | 0  (0.00) | 0  (0.00) | 0  (0.00) | 0  (0.00) | 0  (0.00) | 0  (0.00) | 0  (0.00) | 0  (0.00) | 0  (0.00) |
| **[C]** | 4  (1.13) | 12  (3.92) | 3  (0.78) | 2  (0.55) | 3  (1.26) | 12  (2.71) | 5  (1.15) | 12  (3.40) | 3  (1.26) | 13  (3.77) |
| **[D]** | 1  (0.28) | 2  (0.65) | 1  (0.26) | 1  (0.27) | 0  (0.00) | 0  (0.00) | 3  (0.69) | 4  (1.13) | 0  (0.00) | 1  (0.29) |
| **[E]** | 9  (2.54) | 6  (1.96) | 3  (0.78) | 2  (0.55) | 5  (2.10) | 12  (2.71) | 8  (1.84) | 11  (3.12) | 5  (2.10) | 10  (2.90) |
| **[F]** | 1  (0.28) | 0  (0.00) | 0  (0.00) | 0  (0.00) | 0  (0.00) | 2  (0.45) | 1  (0.23) | 0  (0.00) | 0  (0.00) | 2  (0.58) |
| **[G]** | 16  (4.52) | 24  (7.84) | 11  (2.85) | 8  (2.19) | 14  (5.88) | 15  (3.39) | 18  (4.14) | 18  (5.10) | 14  (5.88) | 15  (4.35) |
| **[H]** | 4  (1.13) | 0  (0.00) | 0  (0.00) | 0  (0.00) | 0  (0.00) | 3  (0.68) | 1  (0.23) | 3  (0.85) | 0  (0.00) | 0  (0.00) |
| **[I]** | 2  (0.56) | 4  (1.31) | 1  (0.26) | 1  (0.27) | 1  (0.42) | 4  (0.90) | 1  (0.23) | 6  (1.70) | 1  (0.42) | 4  (1.16) |
| **[J]** | 10  (2.82) | 3  (0.98) | 31  (8.03) | 32  (8.74) | 1  (0.42) | 3  (0.68) | 4  (0.92) | 3  (0.85) | 1  (0.42) | 3  (0.87) |
| **[K]** | 21  (5.93) | 15  (4.90) | 17  (4.40) | 17  (4.64) | 14  (5.88) | 20  (4.51) | 34  (7.82) | 12  (3.40) | 14  (5.88) | 12  (3.48) |
| **[L]** | 34  (9.60) | 14  (4.58) | 17  (4.40) | 13  (3.55) | 7  (2.94) | 23  (5.19) | 20  (4.60) | 11  (3.12) | 7  (2.94) | 17  (4.93) |
| **[M]** | 21  (5.93) | 31  (10.13) | 35  (9.07) | 27  (7.38) | 26  (10.92) | 33  (7.45) | 32  (7.36) | 24  (6.80) | 27  (11.34) | 24  (6.96) |
| **[N]** | 14  (3.95) | 5  (1.63) | 15  (3.89) | 13  (3.55) | 9  (3.78) | 8  (1.81) | 3  (0.69) | 3  (0.85) | 11  (4.62) | 10  (2.90) |
| **[O]** | 3  (0.85) | 15  (4.90) | 17  (4.40) | 17  (4.64) | 17  (7.14) | 9  (2.03) | 4  (0.92) | 14  (3.97) | 14  (5.88) | 15  (4.35) |
| **[P]** | 8  (2.26) | 2  (0.65) | 7  (1.81) | 7  (1.91) | 2  (0.84) | 5  (1.13) | 9  (2.07) | 2  (0.57) | 1  (0.42) | 3  (0.87) |
| **[Q]** | 1  (0.28) | 0  (0.00) | 0  (0.00) | 0  (0.00) | 0  (0.00) | 0  (0.00) | 2  (0.46) | 1  (0.28) | 0  (0.00) | 0  (0.00) |
| **[T]** | 7  (1.98) | 6  (1.96) | 9  (2.33) | 8  (2.19) | 9  (3.78) | 5  (1.13) | 10  (2.30) | 9  (2.55) | 10  (4.20) | 10  (2.90) |
| **[U]** | 14  (3.95) | 13  (4.25) | 24  (6.22) | 24  (6.56) | 17  (7.14) | 16  (3.61) | 16  (3.68) | 12  (3.40) | 19  (7.98) | 15  (4.35) |
| **[V]** | 6  (1.69) | 1  (0.33) | 1  (0.26) | 2  (0.55) | 3  (1.26) | 4  (0.90) | 10  (2.30) | 1  (0.28) | 4  (1.68) | 2  (0.58) |
| **[W]** | 0  (0.00) | 0  (0.00) | 0  (0.00) | 0  (0.00) | 0  (0.00) | 0  (0.00) | 0  (0.00) | 0  (0.00) | 0  (0.00) | 0  (0.00) |
| **[R], [S],**  **out_COG** | 178  (50.28) | 153  (50.00) | 194  (50.26) | 192  (52.46) | 110  (46.22) | 269  (60.72) | 254  (58.39) | 207  (58.64) | 107  (44.96) | 189  (54.78) |
| **Total** | 354  (100.00) | 306  (100.00) | 386  (100.00) | 366  (100.00) | 238  (100.00) | 443  (100.00) | 435  (100.00) | 353  (100.00) | 238  (100.00) | 345  (100.00) |

^a^: The number in the bracket is the average percentage of corresponding function type in Genome islands by Islandviewer aomong 10 strains.
