# Supplementary material for: High-Density Transcriptional Initiation Signals Underline Genomic Islands in Bacteria
Source: PLoS One. 2012 Mar 20;7(3):e33759. doi: 10.1371/journal.pone.0033759 (PMC3309015; doi:10.1371/journal.pone.0033759)
Supplement: Dataset S10 — Distribution of GIs in different subcellular locations in genomes, and detected by GIST and Islandviewer. (DOC) [file pone.0033759.s013.doc]

| **Strains** | **subcellular location** | | | | | | |
| --- | --- | --- | --- | --- | --- | --- | --- |
|  | Cytoplasmic | Cytoplasmic  Membrane | Extracellular | OuterMembrane | Periplasmic | Unknown | Total |
| **NC_000913** | 1795  (0.4642)^a^ | 1030  (0.2664) | 44  (0.0114) | 75  (0.0194) | 151  (0.039) | 772  (0.1996) | 3867  (1) |
| **NC_003197** | 1942  (0.4391) | 1086  (0.2455) | 69  (0.0156) | 95  (0.0215) | 150  (0.0339) | 1081  (0.2444) | 4423  (1) |
| **NC_003198** | 3819  (0.4362) | 2049  (0.234) | 131  (0.015) | 174  (0.0199) | 278  (0.0318) | 2304  (0.2632) | 8755  (1) |
| **NC_004631** | 1903  (0.4411) | 1022  (0.2369) | 65  (0.0151) | 85  (0.0197) | 136  (0.0315) | 1103  (0.2557) | 4314  (1) |
| **NC_006511** | 1825  (0.4464) | 999  (0.2444) | 63  (0.0154) | 87  (0.0213) | 138  (0.0338) | 976  (0.2387) | 4088  (1) |
| **NC_006905** | 1849  (0.4224) | 1045  (0.2387) | 69  (0.0158) | 86  (0.0196) | 136  (0.0311) | 1192  (0.2723) | 4377  (1) |
| **NC_010067** | 1822  (0.4051) | 1020  (0.2268) | 65  (0.0145) | 93  (0.0207) | 135  (0.03) | 1363  (0.303) | 4498  (1) |
| **NC_010102** | 1957  (0.35) | 1197  (0.2141) | 72  (0.0129) | 98  (0.0175) | 148  (0.0265) | 2120  (0.3791) | 5592  (1) |
| **NC_011147** | 1829  (0.4493) | 988  (0.2427) | 63  (0.0155) | 89  (0.0219) | 138  (0.0339) | 964  (0.2368) | 4071  (1) |
| **NC_012125** | 1880  (0.4117) | 1084  (0.2374) | 64  (0.014) | 93  (0.0204) | 142  (0.0311) | 1303  (0.2854) | 4566  (1) |

^a^: The number in the bracket is the average proportion of corresponding subcellular location in 10 strains.

| **Strains** | **methods** | **subcellular location** | | | | | | |
| --- | --- | --- | --- | --- | --- | --- | --- | --- |
|  |  | Cytoplasmic | Cytoplasmic  Membrane | Extracellular | OuterMembrane | Periplasmic | Unknown | Total |
| NC_000913 | Island-viewer | 77  (0.4231) ^a^ | 27  (0.1484) | 4  (0.022) | 6  (0.033) | 7  (0.0385) | 61  (0.335) | 182  (1) |
|  | GIST | 137  (0.4177) | 65  (0.1982) | 14  (0.0427) | 12  (0.0366) | 11  (0.0335) | 89  (0.271) | 328  (1) |
| NC_003197 | Islandviewer | 104  (0.3086) | 61  (0.181) | 10  (0.0297) | 7  (0.0208) | 5  (0.0148) | 150  (0.445) | 337  (1) |
|  | GIST | 136  (0.3716) | 69  (0.1885) | 16  (0.0437) | 12  (0.0328) | 16  (0.0437) | 117  (0.319) | 366  (1) |
| NC_003198 | Islandviewer | 157  (0.382) | 65  (0.1582) | 12  (0.0292) | 8  (0.0195) | 11  (0.0268) | 158  (0.384) | 411  (1) |
|  | GIST | 128  (0.3422) | 69  (0.1845) | 15  (0.0401) | 13  (0.0348) | 17  (0.0455) | 132  (0.352) | 374  (1) |
| NC_004631 | Islandviewer | 146  (0.3668) | 64  (0.1608) | 13  (0.0327) | 8  (0.0201) | 6  (0.0151) | 161  (0.404) | 398  (1) |
|  | GIST | 116  (0.3779) | 49  (0.1596) | 12  (0.0391) | 9  (0.0293) | 11  (0.0358) | 110  (0.358) | 307  (1) |
| NC_006511 | Islandviewer | 73  (0.312) | 49  (0.2094) | 12  (0.0513) | 8  (0.0342) | 5  (0.0214) | 87  (0.371) | 234  (1) |
|  | GIST | 125  (0.3655) | 57  (0.1667) | 19  (0.0556) | 14  (0.0409) | 12  (0.0351) | 115  (0.336) | 342  (1) |
| NC_006905 | Islandviewer | 134  (0.29) | 65  (0.1407) | 12  (0.026) | 8  (0.0173) | 7  (0.0152) | 236  (0.510) | 462  (1) |
|  | GIST | 130  (0.3308) | 72  (0.1832) | 15  (0.0382) | 13  (0.0331) | 12  (0.0305) | 151  (0.384) | 393  (1) |
| NC_010067 | Islandviewer | 118  (0.2664) | 58  (0.1309) | 19  (0.0429) | 17  (0.0384) | 5  (0.0113) | 226  (0.510) | 443  (1) |
|  | GIST | 116  (0.2959) | 67  (0.1709) | 18  (0.0459) | 17  (0.0434) | 9  (0.023) | 165  (0.420) | 392  (1) |
| NC_010102 | Islandviewer | 102  (0.2849) | 65  (0.1816) | 4  (0.0112) | 5  (0.014) | 6  (0.0168) | 176  (0.491) | 358  (1) |
|  | GIST | 143  (0.2591) | 97  (0.1757) | 16  (0.029) | 15  (0.0272) | 15  (0.0272) | 266  (0.481) | 552  (1) |
| NC_011147 | Islandviewer | 69  (0.304) | 47  (0.207) | 12  (0.0529) | 9  (0.0396) | 4  (0.0176) | 86  (0.378) | 227  (1) |
|  | GIST | 124  (0.3636) | 60  (0.176) | 17  (0.0499) | 16  (0.0469) | 10  (0.0293) | 114  (0.334) | 341  (1) |
| NC_012125 | Islandviewer | 90  (0.2564) | 59  (0.1681) | 8  (0.0228) | 5  (0.0142) | 5  (0.0142) | 184  (0.524) | 351  (1) |
|  | GIST | 127  (0.312) | 84  (0.2064) | 15  (0.0369) | 13  (0.0319) | 11  (0.027) | 157  (0.385) | 407  (1) |

^a^: The number in the bracket is the average proportion of corresponding subcellular location in 10 strains.
