# Supplementary material for: High-Density Transcriptional Initiation Signals Underline Genomic Islands in Bacteria
Source: PLoS One. 2012 Mar 20;7(3):e33759. doi: 10.1371/journal.pone.0033759 (PMC3309015; doi:10.1371/journal.pone.0033759)
Supplement: Dataset S12 — Diagrams showing proposed reasons for enrichments of TSPs in GI regions. (PDF) [file pone.0033759.s015.pdf]

Additional File 10:

The two diagrams of the proposed reasons for why TSPs are enriched in GI regions.

(A), Evolution of regulatory region. To eliminate the fitness cost by non-optimal codon usage or absonant transcripts of GI, accelerated evolution in regulatory regions will result in TSPs accumulation in GI region.

(B), Re-shape of GI region. Following the event of HGT, the GI region might undergo gene loss/acquisition and genetic rearrangements. During this process, former operon was broken and more transcription units probably formed. Newly formed transcription units carry or call for the new TSPs during evolution.

(A)

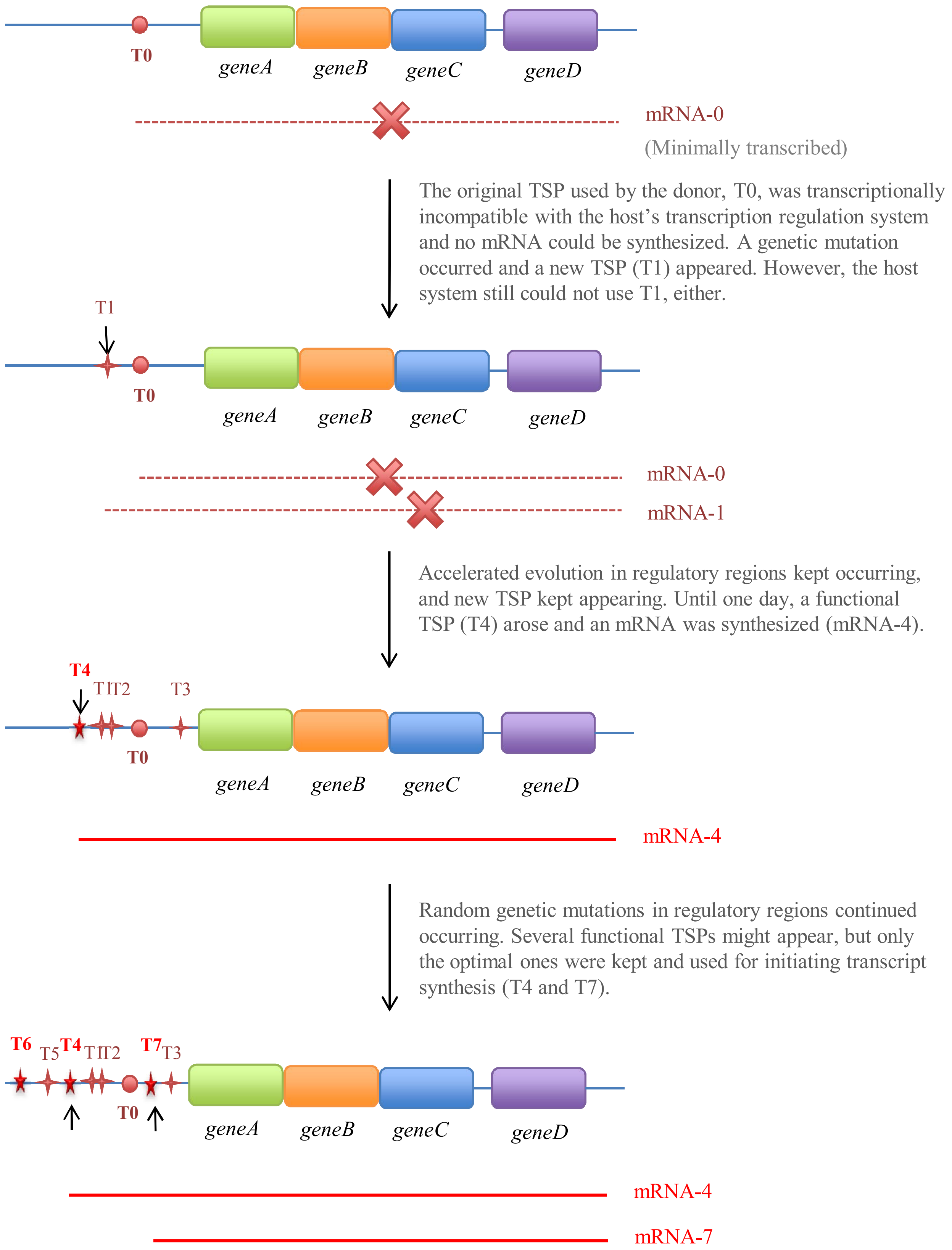
T0
geneA
geneB
geneC
geneD
mRNA-0
(Minimally transcribed)
The original TSP used by the donor, T0, was transcriptionally incompatible with the host's transcription regulation system and no mRNA could be synthesized. A genetic mutation occurred and a new TSP (T1) appeared. However, the host system still could not use T1, either.
T1
T0
geneA
geneB
geneC
geneD
mRNA-0
mRNA-1
Accelerated evolution in regulatory regions kept occurring, and new TSP kept appearing. Until one day, a functional TSP (T4) arose and an mRNA was synthesized (mRNA-4).
T4
T1T2
T3
T0
geneA
geneB
geneC
geneD
mRNA-4
Random genetic mutations in regulatory regions continued occurring. Several functional TSPs might appear, but only the optimal ones were kept and used for initiating transcript synthesis (T4 and T7).
T6
T5
T4
T1T2
T7
T3
T0
geneA
geneB
geneC
geneD
mRNA-4
mRNA-7

(B)

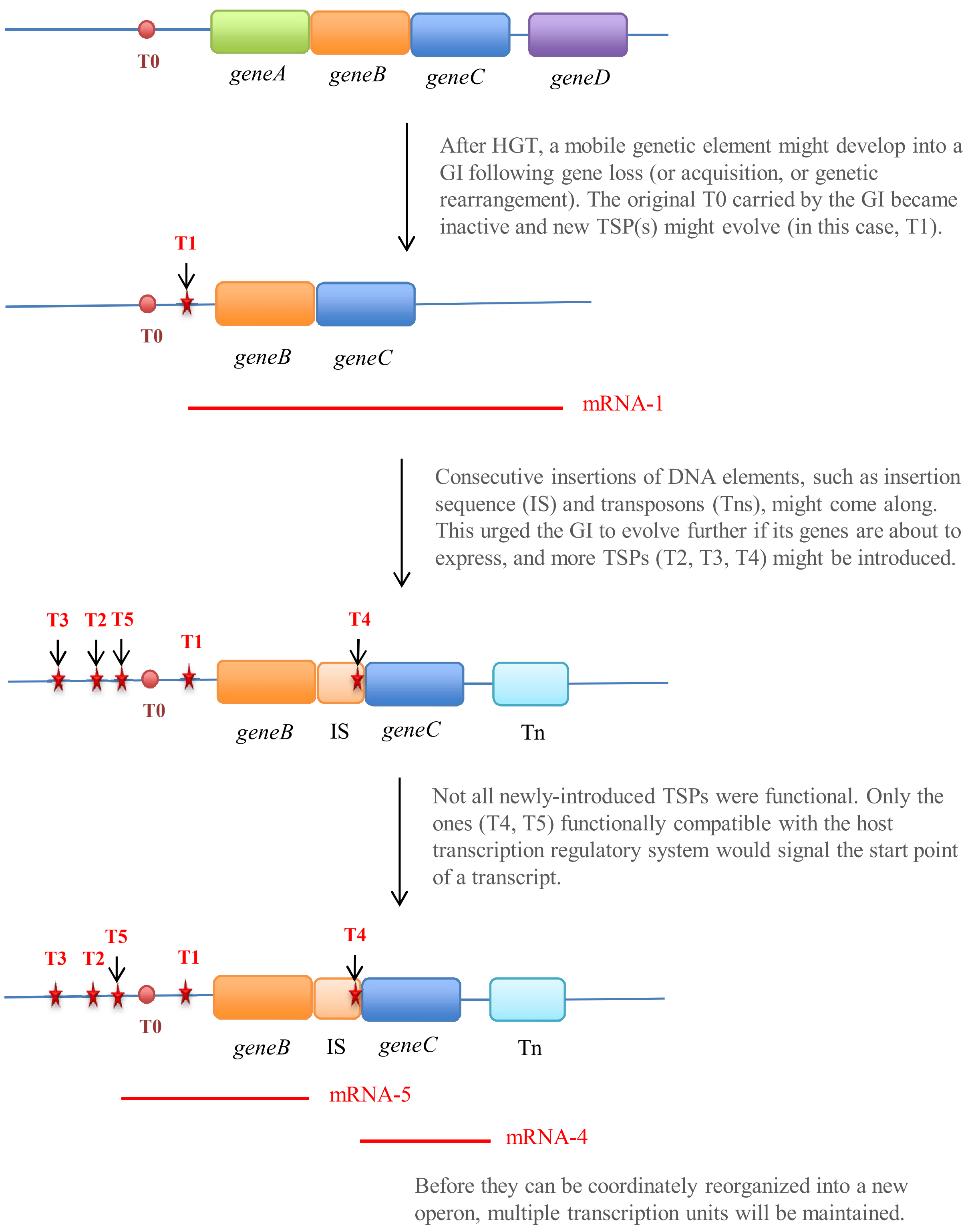

T0
geneA
geneB
geneC
geneD
After HGT, a mobile genetic element might develop into a GI following gene loss (or acquisition, or genetic rearrangement). The original T0 carried by the GI became inactive and new TSP(s) might evolve (in this case, T1).
T1
T0
geneB
geneC
mRNA-1
Consecutive insertions of DNA elements, such as insertion sequence (IS) and transposons (Tns), might come along. This urged the GI to evolve further if its genes are about to express, and more TSPs (T2, T3, T4) might be introduced.
T3
T2
T5
T1
T4
T0
geneB
IS
geneC
Tn
Not all newly-introduced TSPs were functional. Only the ones (T4, T5) functionally compatible with the host transcription regulatory system would signal the start point of a transcript.
T5
T3
T2
T1
T4
T0
geneB
IS
geneC
Tn
mRNA-5
mRNA-4
Before they can be coordinately reorganized into a new operon, multiple transcription units will be maintained.
